# Comparative study of gold and silver interactions with amino acids and nucleobases†

**DOI:** 10.1039/d0ra06486f

**Published:** 2020-09-15

**Authors:** Andrey A. Buglak, Alexei I. Kononov

**Affiliations:** a St. Petersburg State University 199034 Saint-Petersburg Russia andreybuglak@gmail.com

## Abstract

Metal nanoclusters (NCs) have gained much attention in the last decade. In solution, metal nanoclusters can be stabilized by proteins, and, thus, exhibit many advantages in biocatalysis, biosensing, and bioimaging. In spite of much progress in the synthesis of polypeptide-stabilized gold (Au) clusters, their structure, as well as amino acid-cluster and amino acid–Au^+^ interactions, remain poorly understood. It is not entirely clear which amino acid (AA) residues and sites in the protein are preferred for binding. The understanding of NC-protein interactions and how they evolve in the polypeptide templates is the key to designing Au NCs. In this work, binding of gold ion Au^+^ and diatomic neutral gold nanocluster Au_2_ with a full set of α-proteinogenic amino acids is studied using Density Functional Theory (DFT) and the *ab initio* RI-MP2 method in order to find the preferred sites of gold interaction in proteins. We demonstrated that the interaction of gold cations and clusters with protonated and deprotonated amino acid residues do not differ greatly. The binding affinity of AAs to the Au_2_ cluster increases in the following order: Cys(−H^+^) > Asp(−H^+^) > Tyr(−H^+^) > Glu(−H^+^) > Arg > Gln, His, Met ≫ Asn, Pro, Trp > Lys, Tyr, Phe > His(+H^+^) > Asp > Lys(+H^+^) > Glu, Leu > Arg(+H^+^) > Ile, Val, Ala > Thr, Ser > Gly, Cys, which agrees with the available experimental data that gold cluster synthesis occurs in a wide range of pH – amino acid residues with different protonation states are involved in this process. The significant difference in the binding energy of metal atoms with nucleobases and amino acids apparently means that unlike on DNA templates, neutral metal atoms are strongly bound to amino acid residues and can't freely diffuse in a polypeptide globula. This fact allows one to conclude that formation of metal NCs in proteins occurs through the nucleation of reduced Au atoms bound to the neighboring amino acid residues, and the flexibility of the amino acid residue side-chains and protein chain as a whole plays a significant role in this process.

## Abstract 2

Our calculations showed that amino acids stabilize gold nanoclusters; binding energy between organics and gold is higher than between organics and silver.

## Graphical abstract

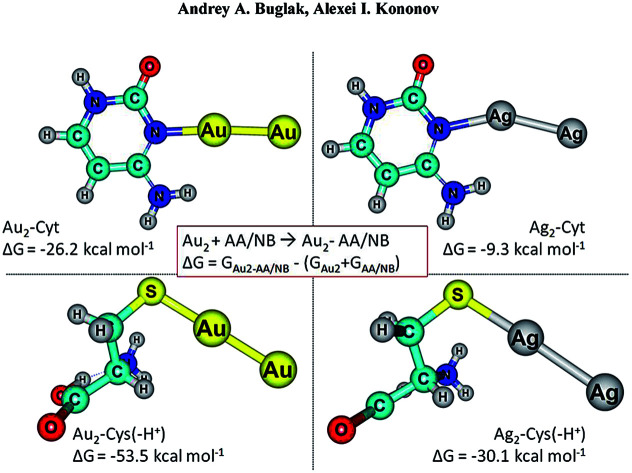

## 1. Introduction

Metal nanoclusters (NCs) have gained much attention in the last decade in biocatalysis, biosensing, and bioimaging due to their high biocompatibility, small size, and high sensitivity to molecular environment.^1–3^ NCs have been used for the detection of metal ions, simple organic compounds, thiols, polypeptides, proteins, and nucleic acids.^4–7^ In solution, NCs can be stabilized by various polymer templates. In particular, DNA-stabilized^8,9^ and protein-protected^6,7^ NCs exhibit many advantages for biosensing and bioimaging: ultrasmall size, photostability, biocompatibility, and brightness. Noble metal NCs, in particular, silver (Ag) and gold (Au) clusters, in comparison with other NCs, exhibit excellent stability, facile synthesis, and low toxicity.^10^ Metal clusters emitting in the visible range have been synthesized using DNA,^8,9^ amino acids,^11,12^ peptides,^13,14^ and proteins such as bovine serum albumin,^15–19^ human serum albumin, egg albumin,^20,21^ lysozyme,^22^ and immunoglobulin.^5^

In spite of much progress in the synthesis of a wide variety of NCs, their structure, as well as ligand–cluster interactions, remains poorly understood. For protein-stabilized NCs, it is not entirely clear which amino acid residues and sites in the polypeptide are preferred for binding. The understanding of NCs–polypeptide interactions and how they evolve in the polypeptide matrices is the key to design the functional fluorescent biolabels. We have investigated earlier the interactions of amino acids with silver ions and clusters.^23^ We showed that deprotonated amino acid residues are preferable for binding with silver clusters, which is in line with experimental data: the formation of silver clusters on protein templates occurs predominantly at alkaline pH. On the contrary, gold clusters are synthesized in a wide range of pH.^24^ Several factors may influence this process: the ability of different amino acid functional groups to reduce gold, charge, and binding energy with gold.

This paper focuses on the Au_2_ cluster/amino acid (AA) interactions and on the Au^+^/AA interactions as a precursor of the cluster. In a typical synthesis, hydrogen tetrachloroaurate is usually taken as a source of gold Au^3+^ ions. At the first stage, proteins reduce most of the Au^3+^ to Au^1+^.^25^ It is generally believed that at the first step of Au NPs synthesis Au^+^–thiolate complexes are formed.^26–28^ In proteins, Au ions may bind to various AAs able to interact with gold. Many AAs can reduce the ions.^29–33^

It is known that Au can interact with O, N and S atoms. At the same time, it is believed that gold in protein templates interacts most effectively with sulfur, especially with cysteine. Indeed, cysteine is considered as the preferential binding site for gold in proteins, which is confirmed by theoretical calculations.^34^

The interaction of gold cation (Au^+^) and gold nanoparticles with individual amino acids and proteins has been investigated earlier.^29–33,35–37^ It was shown experimentally that low temperature and acidic pH favors the growth of gold nanoparticles on protein template.^35^ On contrary, we showed that deprotonated amino acid residues are preferable for binding with silver clusters,^23^ which is in line with experimental data: the formation of silver clusters on protein templates occurs predominantly at alkaline pH.^5,18,38–40^ Gold clusters are synthesized in a wide range of pH.^24,41–44^ The synthesis of gold nanostructures through the photo-reduction of amino acids in water is also possible: the most stable structures are produced by arginine, cysteine, threonine, methionine, tryptophan, and phenylalanine.^36^ The interaction of gold cation (Au^+^), gold clusters, and nanoparticles with individual amino acids and proteins was investigated theoretically earlier.^37,45–47^ Using molecular dynamics, it was shown that negatively charged atoms play a significant role in adsorption of amino acids on the gold nanoparticles.^37^ It was shown that the interaction energy with neutral Au_3_ cluster is higher for the glycine bearing a negative charge than for glycine with charge 0 or +1, the same is true for cysteine.^45^ In the case of neutral and negatively charged glycine, the interaction occurs with the nitrogen atom, while in the case of protonated glycine interaction with Au_3_ cluster proceeds through the carboxyl. In the case of cysteine, neutral amino acid interacts with the cluster through the nitrogen, negatively charged amino acid forms a bond between Au and sulfur, while for positively charged Gly interaction with Au occurs through both sulfur and hydroxyl of the carboxyl group. They also showed that two major bonding factors are: (1) Au–N, Au–O, and Au–S anchoring bonding; and (2) nonconventional OH⋯Au and NH⋯Au hydrogen bonding.^45^ Rai and co-authors performed theoretical calculations for proline and Au_3_ cluster. The interaction of gold cluster with proline occurred predominantly through amide terminal.^46^ Investigation of the interactions of Au_8_ and Au_20_ clusters with alanine and tryptophan showed that these clusters prefer single-site interactions through the amino-group for the amino acids.^47^ In these works, the authors regarded only alanine, cysteine, glycine, proline, and tryptophan interaction with gold nanoclusters and how the interplay of gold with the remaining 15 amino acids occurs was still unknown.

In our quantum-chemical investigation we focused on the interactions of the neutral Au_2_ cluster and Au^+^ cation with a full set of proteinogenic α-amino acids. We tried to identify favorable sites of clusters formation on protein templates. Also, we took into account the effect of amino acid side chain protonation/deprotonation on the effectiveness of these interactions. The Au_2_ cluster was chosen as a model object since it is a minimal singlet cluster with a neutral charge. Au_2_ diatomic cluster protected by organic ligands is a classical object of nanocluster research.^48,49^ We calculated the binding Gibbs free energies between Au_2_ and amino acids in the neutral, protonated, and deprotonated forms of the side chain. We established the amino acids, which are more preferable for the interaction with Au_2_ and Au^+^. Next, we compared gold–amino acid binding energies with silver binding energies. Also, we examined the interactions in the complexes using Bader's quantum theory “Atoms-in-molecules” approach and natural bond orbital (NBO) analysis.

There is a certain interest to compare the binding energies of gold and silver clusters with protein and DNA matrices. It was shown experimentally that both Au and Ag NCs on protein matrices consist mostly of neutral metal atoms.^15–17,22^ In contrast, DNA-stabilized NCs are positively charged clusters.^50–52^ We performed the calculations for gold and silver ions and clusters with cytosine and adenine, the preferred binding residues for Ag and Au clusters in DNA,^53,54^ and compared them with the literature data on Au and Ag interactions with DNA.

## 2. Methods

Equilibrium geometry optimizations and corresponding hessian calculations of complexes were done with usage of density functional theory (DFT) at the PBE^55^ level, and resolution-of-the-identity second order Moller–Plesset perturbation theory (RI-MP2) realized in Orca 3.0 program package.^56^ Since DFT does not include dispersion forces, the atom-pair wise dispersion correction with Becke–Johnson damping was used.^57^ Karlsruhe basis set def2-TZVP was used in all the calculations, gold atoms were treated with def2-TZVP effective core potential (ECP).^58^ The initial geometries of amino acid–Au_2_ complexes for optimization were constructed by placing gold atoms near the active sites of amino acids. The active sites of the amino acids are the amino group, the carboxyl group, and sulfur. These groups possess electron-rich nitrogen, oxygen, and sulfur, which may donate electron density to Au from their lone electron pairs. Binding Gibbs free energies (Δ*G*) were calculated for the reactions:Au^+^ + AA → AA–Au^+^andAu_2_ + AA → AA–Au_2_

The NBO analysis^59^ was performed for the amino acid–Au_2_ complexes using NBO.5 program at the RI-MP2/def2-TZVP level of theory. The NBO analysis was done in order to obtain natural charges and Wiberg bond indices. The NBO orbitals of several complexes were plotted using the Chemcraft program. The atoms-in-molecules (AIM) analysis was performed with the Multiwfn program package^60^ to calculate the properties of bond critical points (BCPs).

## 3. Results and discussion

In our previous study, we showed that RI-MP2 method in combination with def2-TZVP basis set gives reasonable results predicting Gibbs free energy of interaction between simple organic molecules and silver.^61^ In our next study, we showed that PBE-D3 method along with RI-MP2 give fruitful results when calculating Gibbs free energy of interaction between silver and amino acid residues.^23^ Thus, in this study we used both PBE-D3 and RI-MP2 method with def2-TZVP basis set and def2-TZVP ECP for another noble metal–gold.

### 3.1 Amino acid interaction with gold cation Au^+^

We started with the analysis of amino acid interactions with gold cation Au^+^. Among the neutral amino acids, arginine had the highest binding free energy (Δ*G*) with Au^+^: −130.7 kcal mol^−1^ and −140.7 kcal mol^−1^, according to RI-MP2/def2-TZVP and PBE-D3/def2-TZVP, respectively (Table 1). Δ*G* was the lowest one for glycine: −60.1 kcal mol^−1^ and −73.4 kcal mol^−1^, according to RI-MP2/def2-TZVP and PBE-D3/def2-TZVP, respectively. Thus, PBE-D3 tends to overestimate the binding free energy for approximately 10–15 kcal mol^−1^ as compared to RI-MP2, which is considered as a more precise method. For this reason, PBE-D3 method due to its low computational costs was used for the pretreatment of initial amino acid–Au^+^ geometries (attachment to different sites of amino acid and different conformations were compared) while RI-MP2 was used for the precise calculation of the final geometries and Gibbs free energy.

**Table 1:** Gibbs free energies (in kcal mol^−1^) for Au^+^ binding with amino acids calculated using PBE-D3/def2-TZVP and RI-MP2/def2-TZVP method

Amino acid	RI-MP2	PBE-D3
**Amino acids with deprotonated side chain**		
Asp(−H^+^)	−169.8	−183.3
Cys(−H^+^)	−194.1	−207.4
Glu(−H^+^)	−150.0	−166.9
Tyr(−H^+^)	−177.2	−184.3

**Neutral amino acids**		
Ala	−63.3	−76.7
Arg	−130.7	−140.7
Asn	−73.0	−82.8
Asp	−65.4	−79.3
Cys	−74.9	−88.7
Gln	−79.1	−91.2
Glu	−69.0	−85.5
Gly	−60.1	−73.4
His	−93.1	−102.7
Ile	−64.0	−77.4
Leu	−63.5	−78.0
Lys	−116.8	−124.6
Met	−94.8	−107.9
Phe	−82.3	−91.2
Pro	−68.5	−82.3
Ser	−65.1	−75.3
Thr	−65.2	−77.4
Trp	−95.9	−105.3
Tyr	−83.6	−93.8
Val	−63.8	−77.7

**Fig. 1:**
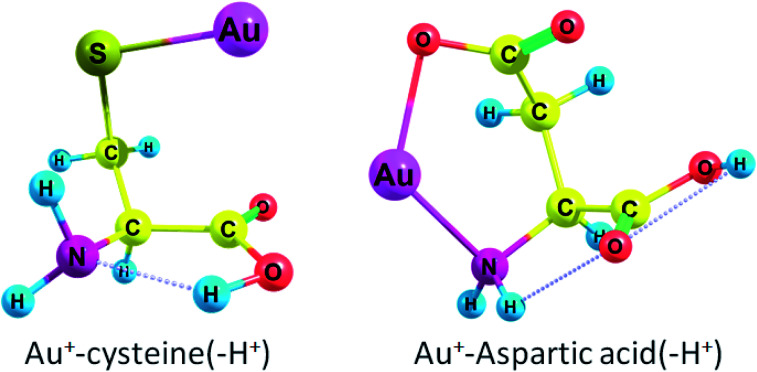
Geometries of deprotonated anionic amino acids bound to gold cation.

Cys(−H^+^) had the highest Δ*G* among all amino acids (see Fig. 1 for complex geometry). Generally, the binding affinity of AAs increases in the following order: Cys(−H^+^) > Tyr(−H^+^) > Asp(−H^+^), Glu(−H^+^) > Arg, Lys > Trp > Met > His > Tyr > Phe > Gln > Cys > Asn > Glu > Pro > Asp > Thr, Ser > Ile, Val, Leu, Ala > Gly. Obviously, we may conclude that Au_2_ will preferably bind to the deprotonated residues of the amino acids rather than to the protonated ones in a peptide or protein. Aliphatic AAs are less preferable for Au^+^ binding. It is also evident that the binding order (preferred binding sites) depends strongly on the pH conditions. Neutral cysteine attaches Au^+^ to both the nitrogen of the amino-group and the sulphur atom while deprotonated through the side-chain anionic cysteine (p*K* = 8.3) forms a monodentate complex with Au^+^ and attaches gold atom solely to the sulphur. Δ*G* rises from −74.9 kcal mol^−1^ (−88.7 kcal mol^−1^) to −194.1 kcal mol^−1^ (−207.4 kcal mol^−1^) during the deprotonation of the cysteine side-chain. Neutral Asp and Glu attach Au^+^ solely to the nitrogen of the amino-group while deprotonated anionic variants of these amino acids attach Au^+^ to the nitrogen and one of the oxygens of the carboxylate group (Fig. 1 and S1†). Δ*G* rises from −65.4 kcal mol^−1^ (−79.3 kcal mol^−1^) to −169.8 kcal mol^−1^ (−183.3 kcal mol^−1^) during the deprotonation of the Asp side-chain (p*K* = 3.7). For Glu (p*K* = 4.3), Δ*G* rises from −69.0 kcal mol^−1^ (−85.5 kcal mol^−1^) to −150.0 kcal mol^−1^ (−166.9 kcal mol^−1^) upon deprotonation of the side-chain. Both neutral Tyr and Tyr(−H^+^) with deprotonated side-chain (p*K* = 10.5) attach Au^+^ to the amino-group and form a cation–pi interaction with the six-membered ring (Fig. S1†). Δ*G* rises from −83.6 kcal mol^−1^ (−93.8 kcal mol^−1^) to −177.2 kcal mol^−1^ (−184.3 kcal mol^−1^) upon deprotonation.

Thereby, all amino acids form either monodentate or bidentate complexes with Au^+^ (see Fig. S1 in ESI†). Every amino acid except deprotonated cysteine forms a bond between Au^+^ and the nitrogen of the amino group. Aliphatic amino acids (except methionine), Asp, Glu, Pro, Ser, and Thr form a monodentate complex with Au^+^. Cys and Met form a bidentate complex while second bond occurs between sulphur and Au. For Arg, His, and Lys, the second site is the nitrogen of the radical side chain; this second site of Au^+^ attraction may become single in a polypeptide since the first site, amino-group, will participate in the formation of a peptide bond. Trp attaches Au^+^ to one of the carbon atoms of the six-membered ring. Phe, Tyr, and Tyr(−H^+^) form a cation–pi interaction between Au^+^ and a six-membered ring. Asn and Gln attach Au^+^ to the carbonyl of the side chain (Fig. S1†). Hence, the Gibbs free energies for the interaction of Au^+^ with Asp, Cys, Glu, Tyr bearing deprotonated side chain radicals appear to be higher than those for the neutral forms of these amino acids (Table 1).

### 3.2 AIM analysis of amino acid complexes with gold cation Au^+^

Next, we used AIM analysis^62^ to study the nature of bonding interactions: amino acid interactions with gold cation Au^+^ were analyzed in terms of electron density and its derivatives. We used several AIM parameters that are presented in Table 2: the density of all electrons (*ρ*(*r*)), the Laplacian of electron density (∇^2^*ρ*(*r*)), the Lagrangian kinetic energy term (*G*(*r*)), the potential energy density (*V*(*r*)), and the energy density (*H*(*r*)).

**Table 2:** Amino acid complexes with Au^+^; bond critical point (BCP) data from AIM analysis

Complex	BCP	*ρ*, hartree	∇^2^*ρ*, hartree	*G*(*r*), hartree	*V*(*r*), hartree	*H*(*r*), hartree	*E*_bond_, kcal mol^−1^
Arg–Au^+^	Au–NH_2_	0.12875	0.46379	0.16682	−0.21769	−0.05087	68.30
Arg–Au^+^	Au–NH	0.14096	0.49987	0.18537	−0.24578	−0.06040	77.11
Cys–Au^+^	Au–SH	0.10009	0.21356	0.08981	−0.12623	−0.03642	39.61
Cys–Au^+^	Au–NH_2_	0.08420	0.31613	0.09839	−0.11776	−0.01936	36.95
Cys(−H^+^)–Au^+^	Au–S	0.13265	0.12066	0.09774	−0.16531	−0.06757	51.87
Gly–Au^+^	Au–NH_2_	0.11226	0.40289	0.13730	−0.17388	−0.03658	54.55
Tyr–Au^+^	Au–C	0.09177	0.19938	0.08127	−0.11269	−0.31425	35.36
Tyr–Au^+^	Au–NH_2_	0.09620	0.35979	0.11642	−0.14289	−0.02647	44.83
Tyr(−H^+^)–Au^+^	Au–C	0.10641	0.18719	0.09018	−0.13357	−0.04338	41.91
Tyr(−H^+^)–Au^+^	Au–NH_2_	0.08936	0.34254	0.10804	−0.13044	−0.02240	40.93

A positive value of ∇^2^*ρ*(*r*) indicates depletion of electronic charge along the bond, which is typical for electrostatic interaction, while a negative value of ∇^2^*ρ*(*r*) indicates that electronic charge is located between the nuclei, which is a feature of electron-sharing and covalent interaction. All bond critical points (BCPs) in Table 2 have a positive ∇^2^*ρ*(*r*) value, which means that all interactions are electrostatic.

The electronic energy density term *H*(*r*) is a sum of kinetic and potential components:

1 *H*(*r*) = *G*(*r*) + *V*(*r*)

The virial theorem states that *G*(*r*) and *V*(*r*) are related to Laplacian through the equation:2
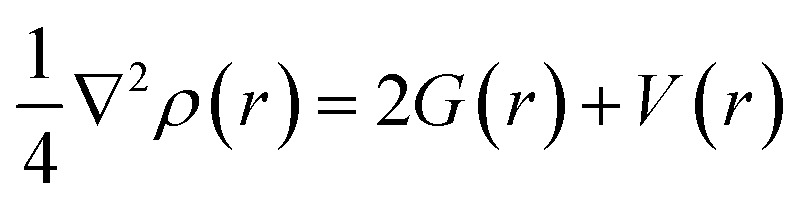


If *H*(*r*) is positive then accumulation of charge at this point is destabilizing. If *H*(*r*) is negative then accumulation of charge is stabilizing. The negative value of *H*(*r*) indicates the presence of a covalent bond. From Table 2 we may see that in all the cases the electrostatic interactions are stabilizing: all *H*(*r*) values are negative. The positive value of ∇^2^*ρ*(*r*) and negative value of *H*(*r*) mean that Au–X bonds are partially covalent and partially electrostatic. That is true for the complexes with both neutral and deprotonated amino acids. For example, AIM parameters calculated for the Au–N bond in the Gly–Au^+^ complex are following: *ρ* = 0.11226 hartree, ∇^2^*ρ* = 0.40289 hartree, and *V*(*r*) = −0.17388 hartree. The *V*(*r*) values allow to calculate the energy of Au–X bond as follows:^63^

3 *E*_bond_ = −*V*(*r*) × 627.51/2

As expected, arginine had the highest Au–X bond energy value (77.1 kcal mol^−1^ for the Au–NH bond) among the regarded complexes. This in line with the Gibbs free energy calculations, which show that Au_2_–Arg complex possesses the highest binding energy among neutral AAs. In Au_2_–Arg complex Au interacts both with the nitrogen of the NH_2_ group (68.3 kcal mol^−1^) and with the NH group of the side chain (for geometry see Fig. S1†). Generally, the deprotonated amino acids had higher bond energy values than the neutral amino acids (this fact is supported by Gibbs free energy calculations): Au–S bond energy was equal to 51.9 kcal mol^−1^ for Au^+^–Cys(−H^+^) and 39.6 kcal mol^−1^ for Au^+^–Cys, Au–C bond energy was equal to 41.9 kcal mol^−1^ for Au^+^–Tyr(−H^+^) and 35.4 kcal mol^−1^ for Au^+^–Tyr.

### 3.3 Interaction of amino acids with Au_2_ cluster

Next, we studied the complexes of amino acids with a minimal neutral cluster Au_2_. In the equilibrium geometry of bare Au_2_, the bond length *r*(Au1–Au2) was equal to 2.465 Å, according to RI-MP2/def2-TZVP method. This bond length tends to slightly diminish in the complexes: for example, *r*(Au1–Au2) was equal to 2.461 Å in Au_2_–Gly.

**Fig. 2:**
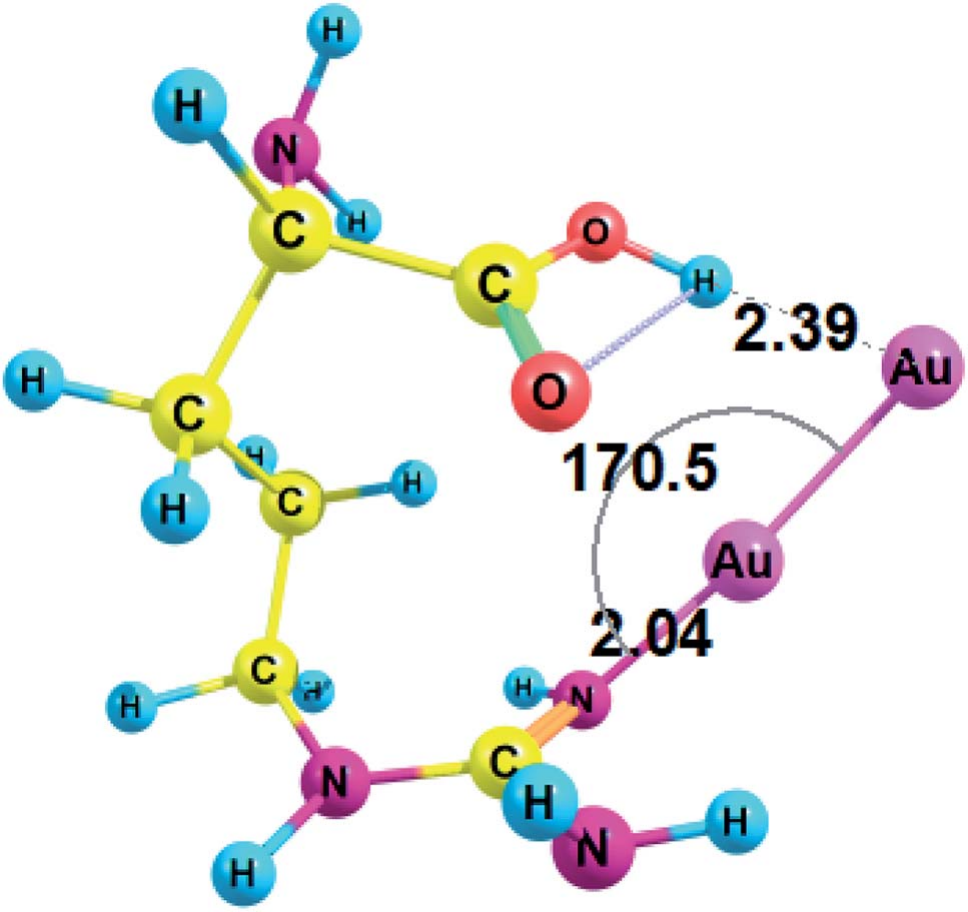
Complex of arginine and Au_2_ cluster optimized with RI-MP2/def2-TZVP method.

Conformations of amino acids were carefully analyzed both free and in complex with Au_2_ (see ESI† for cartesian coordinates). All unionized amino acids attach Au_2_ to the nitrogen of the amino group, except arginine and histidine, which attach Au_2_ to the nitrogen of the side-chain (these interactions between Au NCs and side-chains of amino acid residues may become even more important in a protein since amino group does participate in the formation of a peptide bond). Also, in the case of arginine gold plays the role of a proton acceptor and forms a nonconventional H-bond with the hydroxyl group (O–H⋯Au) of the carboxyl (Fig. 2). Amino acid complexes with gold are always monodentate, except for arginine, which forms a bidentate complex and involves hydrogen bonding. The structural parameters of the complex between arginine and Au_2_ are presented on Fig. 2.

Thus, arginine forms an Au–N coordination bond with a length of 2.040 Å and a valence angle N–Au–Au equal to 170.5° (Fig. 2). For Arg–Au_2_ complex the C–NH bond is increased by 0.028 Å after the interaction with Au_2_ cluster: the C–NH bond is equal to 1.308 for the Arg–Au_2_ complex while for free Arg C–NH bond is equal to 1.280 Å. In addition, the stretching mode of *ν*(C–N) undergoes a blue shift with respect to that of the uncoordinated C–N group: from 1730.5 cm^−1^ to 1635.5 cm^−1^.

**Fig. 3:**
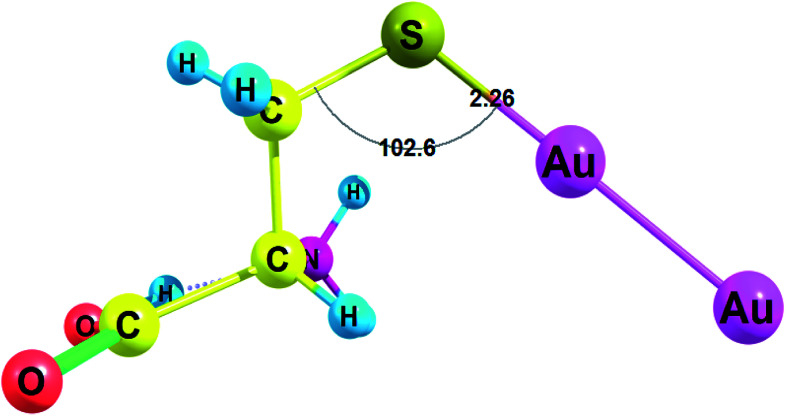
Complex of cysteine(−H^+^) with Au_2_ cluster optimized with RI-MP2/def2-TZVP.

Amino acids with deprotonated side chain surpass Δ*G* values of the neutral amino acids while Cys(−H^+^) possesses the highest Δ*G* among the deprotonated amino acids. Cys(−H^+^) attaches the cluster through the sulfur atom. Cys(−H^+^) forms the Au–S bond with a length of 2.260 Å and a valence angle C–S–Au equal to 102.6° (Fig. 3). For Cys(−H^+^)–Au_2_ complex, the C–S bond stays intact after the interaction with Au_2_ cluster: the C–S bond is equal to 1.821 Å for the Cys(−H^+^)–Au_2_ complex and the same C–S bond is equal to 1.821 Å for the free Cys(−H^+^).

Cys(−H^+^) had the highest Δ*G* among all amino acids (see Fig. 3 for complex geometry). Generally, the binding affinity of AAs to Au_2_ cluster increases in the following order: Cys(−H^+^) > Asp(−H^+^) > Tyr(−H^+^) > Glu(−H^+^) > Arg > Gln, His, Met ≫ Asn, Pro, Trp > Lys, Tyr, Phe > His(+H^+^) > Asp > Lys(+H^+^) > Glu, Leu > Arg(+H^+^) > Ile, Val, Ala > Thr, Ser > Gly, Cys. Obviously, we may conclude that Au_2_ will preferably bind to the deprotonated residues of the amino acids rather than to the protonated ones in a peptide or protein. Surprisingly, neutral cysteine has the lowest binding energy among all the amino acids, which results in the highest Δ*G* difference between the neutral and deprotonated amino acid. Generally, the interaction of Au_2_ with protonated and deprotonated AAs do not differ greatly.

It is generally believed that noble metal atoms preferably interact with sulfur in peptides and amino acids.^45,61^ For cysteine Δ*G* was equal to −19.1 kcal mol^−1^ (−17.7 kcal mol^−1^), and for deprotonated cysteine it was found to be −53.5 kcal mol^−1^ (−50.8 kcal mol^−1^) (Table 3). Cysteine has a p*K*_a_ of 8.3, which means that alkalization of the solution containing Cys and Au_2_ from neutral to alkaline pH would give the increase in interaction energy equal to 34.4 kcal mol^−1^ (33.1 kcal mol^−1^). The interaction energy of Au_2_ with methionine, which also occurs through the sulfur, was equal to −28.1 kcal mol^−1^ (−24.5 kcal mol^−1^), which was one of the highest Δ*G* among the neutral amino acids. The disulfide bond is less favorable for the formation of a complex with the gold cluster: the energy of the interaction between dimethyldisulfide and Au_2_ was equal to −23.4 kcal mol^−1^, according to RI-MP2/def2-TZVP method (for geometry of the complex see Fig. S2†); it was equal to −22.7 kcal mol^−1^, according to the PBE-D3/def2-TZVP calculation.

**Table 3:** Gibbs free energies (in kcal mol^−1^) for amino acid binding with Au_2_ cluster calculated using PBE-D3/def2-TZVP and RI-MP2/def2-TZVP method

Amino acid	RI-MP2	PBE-D3
**Amino acids with deprotonated side chain**		
Asp(−H^+^)	−41.1	−40.6
Cys(−H^+^)	−53.5	−50.8
Glu(−H^+^)	−35.4	−33.5
Tyr(−H^+^)	−39.4	−36.6

**Neutral amino acids**		
Ala	−20.6	−17.9
Arg	−34.3	−30.7
Asn	−26.9	−21.5
Asp	−22.7	−21.1
Cys	−19.1	−17.7
Gln	−28.5	−23.1
Glu	−22.1	−18.2
Gly	−19.2	−17.2
His	−28.3	−26.7
Ile	−21.0	−17.9
Leu	−22.1	−20.7
Lys	−23.3	−19.7
Met	−28.1	−24.5
Phe	−22.9	−21.0
Pro	−26.4	−21.5
Ser	−19.4	−14.3
Thr	−20.6	−17.7
Trp	−25.9	−20.7
Tyr	−23.1	−21.2
Val	−21.0	−18.3

**Amino acids with protonated side chain**		
Arg(+H^+^)	−22.0	−16.8
His(+H^+^)	−22.9	−17.1
Lys(+H^+^)	−22.2	−17.5

Deprotonated anionic amino acids Asp(−H^+^), Glu(−H^+^), and Tyr(−H^+^) attach Au_2_ to the side chain namely to the oxygen atom and have high Δ*G* values: −41.1 kcal mol^−1^ (−40.6 kcal mol^−1^), −35.4 kcal mol^−1^ (−33.5 kcal mol^−1^), and −39.4 kcal mol^−1^ (−36.8 kcal mol^−1^), respectively (Table 3). Tyrosine, which has a p*K*_a_ of 10.5, has a much lower Δ*G* equal to −23.1 kcal mol^−1^ (−21.2 kcal mol^−1^) in the unionized form and that means that alkalization of the solution with Tyr and Au_2_ from neutral to alkaline pH would give an increase in the interaction energy equal to 16.3 kcal mol^−1^ (15.6 kcal mol^−1^). These data show why alkalization of the solution with the protein containing Tyr residues is advantageous when a gold cluster is formed.

Histidine (p*K*_a_ 6.0) has Δ*G* equal to −28.3 kcal mol^−1^ (−26.7 kcal mol^−1^) in the unionized form and −22.9 kcal mol^−1^ (−17.1 kcal mol^−1^) with the protonated side chain. Alkalization of the solution with His and Au_2_ from acid to alkaline and neutral pH would give the increase of interaction energy equal to 5.4 kcal mol^−1^ (9.6 kcal mol^−1^). For lysine (p*K*_a_ 10.5) Δ*G* rises from −22.2 kcal mol^−1^ (−17.5 kcal mol^−1^) for the cationic amino acid with protonated side chain to −23.3 kcal mol^−1^ (−19.7 kcal mol^−1^) for the neutral molecule. For arginine (p*K*_a_ 12.5) Δ*G* changes from −22.0 kcal mol^−1^ (−16.8 kcal mol^−1^) for the cationic form with the protonated side-chain to −34.3 kcal mol^−1^ (−30.7 kcal mol^−1^) for the unionized molecule.

In general, our results are in agreement with experimental results showing that gold nanoparticles have the highest binding affinity with the peptides containing Cys, His, Met, and Tyr residues.^33^ In real experimental conditions, charge repulsion between negatively charged residues Asp(−H^+^), Glu(−H^+^) and chloroaurate anions frustrate the reduction reaction. However, some experimental protocols allow Asp and Glu residues to play significant role in the synthesis of gold nanoclusters.^24^ Moreover, the stability of Au_2_ complexes of all neutral and protonated amino acid residues is rather high (Δ*G* is more than −20 kcal mol^−1^ in absolute values), which indicates that all 20 proteinogenic AAs can stabilize gold NCs.

### 3.4 AIM and natural bond orbital (NBO) analysis of amino acid complexes with Au_2_ cluster

We used Bader's AIM analysis to study the nature of amino acid interactions with Au_2_ cluster in terms of electron density and its derivatives: the density of all electrons *ρ*(*r*), the Laplacian of electron density ∇^2^*ρ*(*r*), the Lagrangian kinetic energy term *G*(*r*), the potential energy density *V*(*r*), and the energy density *H*(*r*) are presented in Table 4.

**Table 4:** Amino acid complexes with Au_2_; bond critical point (BCP) data from AIM analysis

Complex	BCP	*ρ*, hartree	∇^2^*ρ*, hartree	*G*(*r*), hartree	*V*(*r*), hartree	*H*(*r*), hartree	*E*_bond_, kcal mol^−1^
Arg–Au_2_	Au1–NH	0.12082	0.46238	0.15834	−0.20108	−0.04274	63.09
Arg–Au_2_	Au2⋯HO	0.02065	0.04638	0.01193	−0.01226	−0.00033	3.85
Cys–Au_2_	Au–SH	0.11312	0.25384	0.11014	−0.15682	−0.04668	49.20
Cys(−H^+^)–Au_2_	Au–S	0.11773	0.18658	0.09891	−0.15117	−0.05226	47.43
Gly–Au_2_	Au–NH_2_	0.10571	0.42161	0.13766	−0.16992	−0.03226	53.31
Tyr–Au_2_	Au–NH_2_	0.10946	0.43010	0.14239	−0.17726	−0.03487	55.61
Tyr(−H^+^)–Au_2_	Au–O	0.11164	0.52169	0.16384	−0.19725	−0.03342	61.89

One can see that in each case the electrostatic interaction is stabilizing since for each complex *H*(*r*) value is negative. The positive value of ∇^2^*ρ*(*r*) and negative value of *H*(*r*) in all cases means that Au–X bonds are partially covalent and partially electrostatic.

Since we know *V*(*r*) values we can calculate bond energies. Arginine had the highest Au–X bond energy value equal to 63.1 kcal mol^−1^ (Table 4), and this fact is supported by Gibbs energy calculations, which state that arginine has the highest binding energy among neutral AAs. In the Arg–Au_2_ complex, gold interacts both with the nitrogen of the side chain and with hydrogen of the carboxyl group. The hydrogen bond energy is pretty small – 3.9 kcal mol^−1^, but it is a reasonable value for a hydrogen bond.^36^

Next, we performed the NBO analysis. Natural charges of gold atom and amino acid atom to which the cluster is attached are presented in Table 5 (*q*_Au_ and *q*_X_, respectively). When Au and X both have positive charges (the case of Cys–Au_2_ complex), it obliquely indicates that Au–X bond has a covalent nature. When Au and X have opposite charges (all other cases), it indicates that Au–X bond has an electrostatic nature. In all the cases Au_2_ cluster had a negative charge (*q*_cluster_), which means that it oxidizes the coordinated amino acid. The bond orders were evaluated by using Wiberg's bond indices, which are presented in Table 5. The Wiberg bond indices were higher for the deprotonated negatively charged amino acid complexes with Au_2_ than for the neutral complexes: *W*_Au1–X_ is equal to 0.366 for Cys–Au_2_ and is 0.580 for Cys(−H^+^)–Au_2_, W_Au1–X_ is equal to 0.206 for Tyr–Au_2_ and is 0.247 for Tyr(−H^+^)–Au_2_. On contrary, the Au–Au bond order is lower for deprotonated amino acids.

**Table 5:** Calculated natural population analysis (NPA) charges and Wiberg bond indices of the optimized structures of amino acid–Au_2_ complexes

Complex	Bond type	*W*_Au1–X_	*W*_Au1–Au2_	*q*_X_	*q*_Au_	*q*_cluster_
Au_2_	—	—	1.016	—	0	0
Arg–Au_2_	Au1–NH	0.272	0.851	−0.954	0.130	−0.114
Arg–Au_2_	Au2⋯HO	0.055	0.851	0.528	−0.242	−0.114
Cys–Au_2_	Au1–SH	0.366	0.889	0.078	0.024	−0.183
Cys(−H^+^)–Au_2_	Au1–S	0.580	0.756	−0.444	0.038	−0.374
Gly–Au_2_	Au1–NH_2_	0.190	0.918	−0.861	0.090	−0.110
Tyr–Au_2_	Au1–NH_2_	0.206	0.922	−0.862	0.073	−0.124
Tyr(−H^+^)–Au_2_	Au1–O	0.247	0.806	−0.888	0.250	−0.152

NBO analysis gives useful information when analyzing intramolecular bonding and interaction between bonds. The electron donor orbital and electron acceptor orbital occupancies, as well as the interacting stabilization energy *E*(2) obtained from the second-order perturbation theory analysis, are reported in Table 6. The larger the *E*(2) value, the more intensive the interaction between i electron donor orbital and j electron acceptor orbital, which means the more donating tendency from electron donor to electron acceptor. Delocalization of the electron density from occupied bonds or lone pair NBO orbitals to formally unoccupied antibond NBO orbitals indicates a stabilizing donor–acceptor interaction. The intramolecular interaction is formed by the orbital overlap between n, n*, σ and σ* bond orbitals, which leads to the intramolecular charge transfer (ICT) permitting the stabilization of the system. These interactions are observed as an increase in the electron density in anti–bonding orbital that weakens the respective bonds.

**Table 6:** Second order perturbation theory analysis of Fock matrix in NBO basis for some selected amino acid–Ag_2_ complexes

Complex	Donor (i)	Type of orbital	Occupancy	Acceptor (j)	Type of orbital	Occupancy	*E*(2), kcal mol^−1^	*q*_CT_
Arg–Au_2_	N	n	1.815	Au	n*	0.095	44.90	0.060
Cys–Au_2_	Au–Au	σ	1.830	Au–S	σ*	0.176	113.84	0.220
Cys(−H^+^)–Au_2_	Au–Au	σ	1.896	Au–S	σ*	0.120	65.05	0.121
Gly–Au_2_	N	n	1.859	Au1–Au2	σ*	0.086	39.32	0.058
Tyr–Au_2_	N	n	1.849	Au1–Au2	σ*	0.086	36.29	0.052
Tyr(−H+)–Au_2_	O	n	1.826	Au1–Au2	σ*	0.091	37.78	0.057

For each i donor orbital and j acceptor orbital, the stabilization energy *E*(2) of the i → j delocalization was calculated according to the following formula:4
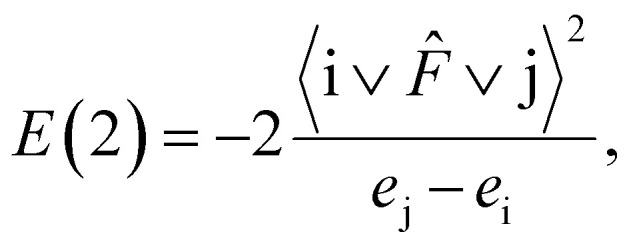
where *e*_i_ and *e*_j_ are NBO orbital energies, and *F̂* is the Fock operator.

The amount of transferred charge from i donor orbital to j acceptor orbital was also calculated using the Fock operator and NBO orbital energies as follows:5
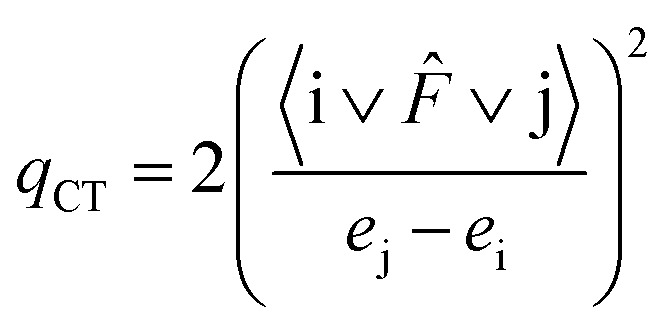


**Fig. 4:**
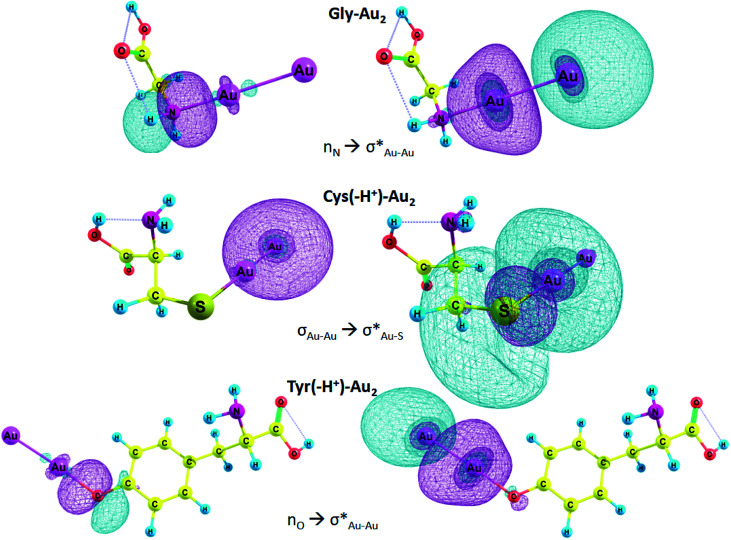
Selected NBO orbitals involved in charge transfer.

In Table 6, *E*(2) and *q*_CT_ for the Au–X and Au–Au bonds of some complexes are presented. The charge is transferred from the lone pair of nitrogen and oxygen or σ Au–Au bond to n* orbital of Au, Au–S or Au–Au σ* anti-bond. In the case of Arg–Au_2_ complex, the charge is transferred from the nitrogen lone pair to the lone pair of Au. In the Gly–Au_2_ complex, the charge is transferred from the nitrogen lone pair to Au–Au σ* anti-bonding orbital (Fig. 4).

We focused on the complexes of cysteine and tyrosine with the gold nanocluster since deprotonation of these amino acids gives the strongest energy gain when the AA–Au_2_ complex is formed (Table 3). Moreover, only these two amino acids exhibited capability to produce fluorescent complexes with silver clusters, as it was show experimentally.^61^ In the Cys–Au_2_ complex, the intramolecular charge transfer occurs from the σ Au–Au bond to Au–S σ* anti-bonding orbital and leads to delocalization of 113.8 kcal mol^−1^, which is the strongest stabilization energy among the regarded complexes. For the Cys(−H^+^)–Au_2_, the same ICT contributes to the stabilization energy of 65.1 kcal mol^−1^. In the Tyr–Au_2_ complex, ICT of the electron from the nitrogen lone pair to the σ* Au–Au anti-bond leads to delocalization of 36.3 kcal mol^−1^. In the Tyr(−H^+^)–Au_2_ complex, ICT of the n electron of oxygen lone pair to the σ* Au–Au anti-bond leads to delocalization equal to 37.8 kcal mol^−1^.

### 3.5 Binding energies between nucleobases and metals

We calculated the interaction energies of metal cations (Ag^+^ and Au^+^), neutral metal atoms (Ag0 and Au0), and diatomic metal clusters (Ag_2_ and Au_2_) with nucleobases, namely cytosine (Cyt) and adenine (Ade). Later, we compared the interaction energies between silver and gold, between different types of metal particles, between nucleobases and selected amino acids (Table 7). Our results are consistent with previously reported data for the nucleobases. Thus, for example, the interaction energy of silver atom Ag0 and cytosine was equal to −3.5 kcal mol^−1^, according to Gwinn with co-authors.^53^ The same complex interaction energy was equal to −7.0 kcal mol^−1^, according to Volkov *et al.*^64^ In our case the result was −8.3 kcal mol^−1^. The advantage of our work is that the vibrational spectra were calculated and Gibbs free energies of complex formation were determined along with Δ*E*_total_. Thus, for Ag0 complex with cytosine Δ*G* is equal to −1.3 kcal mol^−1^. We have also investigated the complexes of Au_2_ with adenine and protonated cytosine; for the latter it was done for the first time.

**Table 7:** Gibbs free energy (*G*) and total energy (*E*) in kcal mol^−1^ for metal binding with amino acids and nucleobases; the table contains both literature data and results calculated in this study with RI-MP2/def2-TZVP method

Complex	Bond	Δ*G*	Δ*E*
**Nucleobases**			
Ag^+^_Cyt	Ag–N1, Ag–O		−50.1 (ref. 64)
	Ag–N1, Ag–O	−55.2	−64.0
Ag0_Cyt	Ag–N1		−3.5 (ref. 53)
	Ag–O		−7.0 (ref. 64)
	Ag–N1	−1.3	−8.3
	Ag–O	2.2	−5.4
Ag_2__Cyt	Ag–N1		−11.5 (ref. 53)
	Ag–O		−22.7 (ref. 64)
	Ag–N1	−9.3	−21.3
	Ag–O	−4.9	−16.6
Au^+^_Cyt	Au–N1	−74.3	−83.6
	Au–O	−67.9	−77.0
Au0_Cyt	Au–N1	−5.7	−13.4
	Au–O	0.3	−6.9
Au_2__Cyt	Au–N1		−24.2 (ref. 65)
	Au–N1	−26.2	−38.2
	Au–O	−14.9	−27.4
Ag0_Cyt(+H^+^)	Ag⋯HN1	1.9	−5.9
Ag_2__Cyt(+H^+^)	Ag–O, Ag⋯HN1	−6.1	−15.9
Au0_Cyt(+H^+^)	Au⋯HN1	1.1	−5.7
Au_2__Cyt(+H^+^)	Au–O, Au⋯HN1	−5.0	−16.9
Ag^+^_Ade	Ag–N7, Ag–NH_2_	−43.2	−52.9
Ag0_Ade	Ag–N7	0.1	−6.9
Ag_2__Ade	Ag–N7	−7.8	−16.9
Au^+^_Ade	Au–N7	−62.6	−71.4
Au0_Ade	Au–N7	−3.9	−13.0
Au_2__Ade	Au–N7	−25.3	−38.3

**Amino acids**			
Ag^+^_Cys(−H^+^)	Ag–S	−151.8 (ref. 23)	−162.0
Ag0_Cys(−H^+^)	Ag–S	−16.7 (ref. 23)	−26.1
Ag_2__Cys(−H^+^)	Ag–S	−30.1 (ref. 23)	−43.1
Au^+^_Cys(−H^+^)	Au–S	−194.1	−204.3
Au0_Cys(−H^+^)	Au–S	−27.6	−36.9
Au_2__Cys(−H^+^)	Au–S	−53.5	−68.0
Ag^+^_Asp(−H^+^)	Ag–O	−152.8 (ref. 23)	−163.6
Ag0_Asp(−H^+^)	Ag–O	−15.3 (ref. 23)	−24.2
Ag_2__Asp(−H^+^)	Ag–O	−30.7 (ref. 23)	−42.3
Au^+^_Asp(−H^+^)	Au–O	−169.8	−180.9
Au0_Asp(−H^+^)	Au–O	−16.9	−26.2
Au_2__Asp(−H^+^)	Au–O	−41.1	−54.5

Yet, we didn't limit our study to only Me–N bonding, we also analyzed the interactions between metal atoms and the carbonyl oxygen of the cytosine. However, Me–O binding energies concede to Me–N interactions. Thus, for example, in Ag0_Cyt complex Δ*G* for the Ag–N binding is equal to −1.3 kcal mol^−1^, while for Ag–O binding Δ*G* is only 2.2 kcal mol^−1^ (Table 7), so it's highly likely that Ag–O bond doesn't occur. The same is true for Au0_Cyt complex: Δ*G* for Au–O interaction is also positive: 0.3 kcal mol^−1^.

**Fig. 5:**
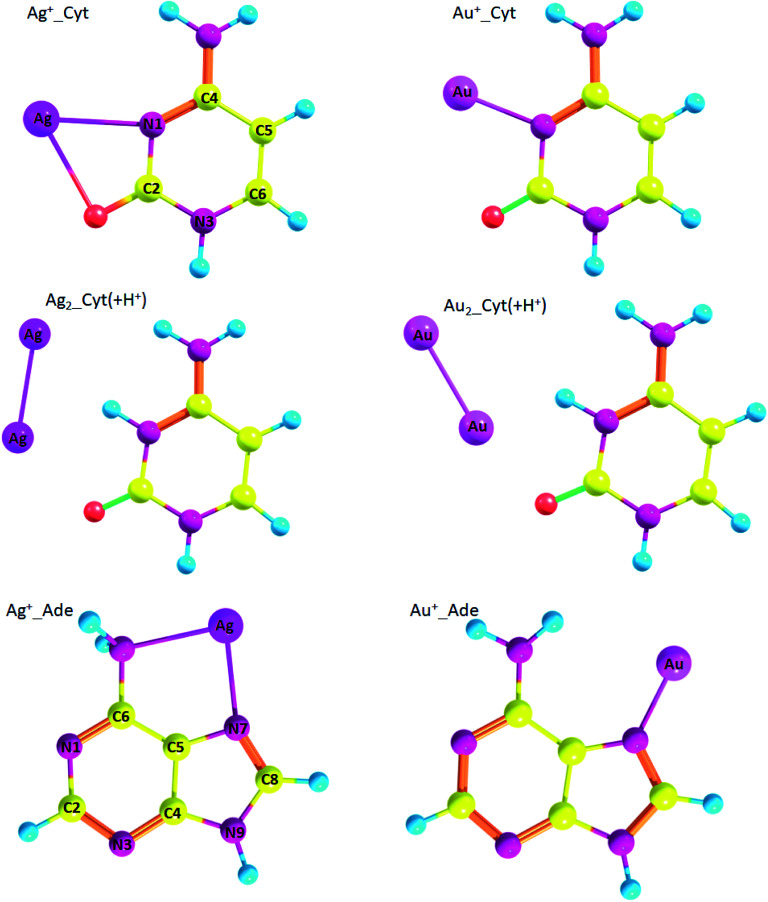
Geometry of metal/nucleobase complexes (optimized at RI-MP2/def2-TZVP level of theory) with atom numbering.

Geometry of the complexes between gold and silver ions and nucleobases, namely cytosine and adenine, are presented on Fig. 5. Ag^+^_Cyt and Au^+^_Cyt complexes have certain differences: the silver ion forms bonds with both nitrogen and oxygen while gold ion attaches only to N1. The same is true for Ag^+^_Ade and Au^+^_Ade complexes: Ag^+^ forms bonds with N7 of the imidazole ring and nitrogen of the amino-group, Au^+^ forms a bond only with N7. Δ*G* binding energy is higher for Au^+^_Cyt than for Ag^+^_Cyt complex: −74.3 kcal mol^−1^ and −55.2 kcal mol^−1^, respectively. Au^+^_Cyt complex with Au–O bonding is less stable than the complex with Au–N bond: −67.9 kcal mol^−1^ and −74.3 kcal mol^−1^, respectively (Table 7). Once again it shows that Me–N bonding is more stable than Me–O bonding.

Speaking about adenine, complexes with gold are more stable than silver complexes. Thus, for Ag^+^_Ade, Ag0_Ade, and Ag_2__Ade Δ*G* is equal to −43.2 kcal mol^−1^, 0.1 kcal mol^−1^, and −7.8 kcal mol^−1^, respectively, while for Au^+^_Ade, Au0_Ade, and Au_2__Ade Δ*G* is equal to −62.6 kcal mol^−1^, −3.9 kcal mol^−1^, and −25.3 kcal mol^−1^ (Table 7), respectively.

Also, we analyzed silver and gold complexes of protonated cytosine (Cyt(+H^+^)). Interestingly, Cyt(+H^+^) tends to form nonconventional hydrogen bonds with metal atoms Me0 and diatomic clusters (Fig. 5). However, Ag0_Cyt(+H^+^) and Au0_Cyt(+H^+^) complexes have positive Δ*G* values and highly likely are not formed: 1.9 kcal mol^−1^ and 1.1 kcal mol^−1^, respectively. Δ*G* values are negative for Ag_2__Cyt(+H^+^) and Au_2__Cyt(+H^+^) complexes: −6.1 kcal mol^−1^ and −5.0 kcal mol^−1^, respectively. The latter complexes are stabilized by Me–O bonds, as well as by nonconventional Me⋯H hydrogen bonding.

We compared metal complexes of nucleobases and amino acids. In all the cases amino acid complexes are more stable than analogous nucleobase complexes. Thus, for example, the most stable complex of Au^+^ is with cytosine (−74.3 kcal mol^−1^) while the binding energy of deprotonated cysteine with Au^+^ is equal to −194.1 kcal mol^−1^. Probably, the reason is that negative charge of the amino acid residue is favorable for high Δ*G*. For the neutral Cys, Δ*G* is comparable with Cyt: −74.9 kcal mol^−1^ (see Table 1).

The interaction of neutral metal atoms and diatomic clusters with AAs is high for both silver and gold. It is known that aspartic acid residues may play a significant role in the growth of protein-templated gold nanoclusters.^24^ For this reason the binding energy between gold and Asp are also high: for Au^+^, Au0, and Au_2_ Δ*G* is equal to −169.8 kcal mol^−1^, −16.9 kcal mol^−1^, and −41.1 kcal mol^−1^.

### 3.6 AIM analysis of metal complexes with cytosine

We used Bader's AIM analysis to study the nature of nucleobase interactions with metal ions, atoms, and nanoclusters in terms of electron density and its derivatives: *ρ*(*r*), ∇^2^*ρ*(*r*), *G*(*r*), *V*(*r*), and *H*(*r*) are presented in Table 8. Metal/nucleobase complex energy density *H*(*r*) value is negative for most of the complexes, which means that the electrostatic interaction between metal atoms and cytosine is stabilizing. The positive value of the Laplacian of electron density ∇^2^*ρ*(*r*) and negative value of *H*(*r*) in most of the cases means that Me–X bonds are partially covalent and partially electrostatic. In the case of Ag0_Cyt(+H^+^) with Ag⋯HN1 interaction and Ag_2__Cyt(+H^+^) with Ag–O interaction the local kinetic energy *G*(*r*) outweighs V(*r*): internuclear charge concentration is destabilizing, which is typical for a nonbonded situation, which in the case of Ag0_Cyt(+H^+^) is also confirmed by positive Δ*G* value (1.9 kcal mol^−1^).

**Table 8:** Cytosine complexes with Au and Ag; bond critical point (BCP) data from AIM analysis

Complex	BCP	*ρ*, hartree	∇^2^*ρ*, hartree	*G*(*r*), hartree	*V*(*r*), hartree	*H*(*r*), hartree	*E*_bond_, kcal mol^−1^
Ag^+^_Cyt	Ag–N1	0.05707	0.25983	0.07118	−0.07741	−0.00622	24.29
Ag^+^_Cyt	Ag–O	0.04822	0.24258	0.06458	−0.06852	−0.00394	21.50
Au^+^_Cyt	Ag–N1	0.13522	0.46462	0.17029	−0.22442	−0.05413	70.41
Ag0_Cyt	Ag–N1	0.05308	0.24042	0.06496	−0.06981	−0.00485	21.90
Au0_Cyt	Au–N1	0.08761	0.35086	0.10845	−0.12919	−0.02074	40.53
Ag_2__Cyt	Ag–N1	0.07269	0.35332	0.09808	−0.10784	−0.00975	33.84
Au_2__Cyt	Au–N1	0.11349	0.46240	0.15277	−0.18995	−0.03718	59.60
Ag0_Cyt(+H^+^)	Ag⋯HN1	0.00866	0.01478	0.00355	−0.00339	0.00015	1.06
Au0_Cyt(+H^+^)	Au⋯HN1	0.02276	0.05869	0.01501	−0.01535	−0.00034	4.82
Ag_2__Cyt(+H^+^)	Ag⋯HN1	0.01995	0.03969	0.01041	−0.01090	−0.00049	3.42
Ag_2__Cyt(+H^+^)	Ag–O	0.02725	0.13028	0.03252	−0.03246	0.00005	10.18
Au_2__Cyt(+H^+^)	Au⋯HN1	0.03923	0.09874	0.03033	−0.03598	−0.00565	11.29
Au_2__Cyt(+H^+^)	Au–O	0.05692	0.28276	0.07701	−0.08333	−0.00632	26.15

Ag^+^_Cyt complex attracted our interest since silver cation forms bonds with both nitrogen and oxygen atoms while Au^+^ forms a bond only with N1 atom of cytosine. The higher energy of potential energy density for Au–N BCP results in the higher energy of the bond equal to 70.4 kcal mol^−1^ as compared with Ag–N (24.3 kcal mol^−1^) and Ag–O (21.5 kcal mol^−1^), which is supported by Gibbs energy calculations (see Table 7). In the case of neutral metal atoms the situation is similar: for Ag0_Cyt complex Ag–N bond energy is equal to 21.6 kcal mol^−1^ while Au–N bond energy in Au0_Cyt complex is much higher: 40.53 kcal mol^−1^. The same is true for Ag_2__Cyt and Au_2__Cyt complexes: bond energies are equal to 33.8 kcal mol^−1^ and 59.6 kcal mol^−1^, respectively. These data are in agreement with Gibbs free energies for metal binding with nucleobases (Table 7): the binding energies are higher for gold atoms than for silver.

## 4. Conclusion

Binding energies between 19 amino acids and gold nanoparticles have been studied previously using molecular dynamics.^66^ In this study, Gibbs free energies of interaction of gold cation Au^+^ and diatomic neutral Au_2_ cluster with the full set of proteinogenic α-amino acids have been calculated by two methods: RI-MP2 and PBE-D3. The complexes of Au^+^ and Au_2_ with deprotonated side chains of all 20 amino acids exhibit the higher values of Δ*G* than that with the neutral and protonated amino acids, which is in agreement with the data obtained earlier for triatomic gold nanocluster Au_3_ complexes with glycine and cysteine.^45^ However, the stability of Au_2_ complexes with neutral and protonated amino acid residues is also rather high (Δ*G* is more than −20 kcal mol^−1^ in absolute values), which indicates that practically all 20 amino acids can stabilize gold NCs and nanoparticles. The binding affinity of AAs to the Au_2_ cluster increases in the following order: Cys(−H^+^) > Asp(−H^+^) > Tyr(−H^+^) > Glu(−H^+^) > Arg > Gln, His, Met > Asn, Pro, Trp > Lys, Tyr, Phe > His(+H^+^) > Asp > Lys(+H^+^) > Glu, Leu > Arg(+H^+^) > Ile, Val, Ala > Thr, Ser > Gly, Cys. Generally, the interaction of gold atoms with protonated and deprotonated amino acid residues do not differ greatly, which is in agreement with the experimental evidences that gold cluster synthesis occurs in a wide range of pH.^24,67^ The fact that deprotonated cysteine has the highest binding energy with both Au_2_ and Au^+^ among all the amino acids explains the fine synthesis of gold nanoclusters on thiolates.^68,69^

Our results suggest that binding energy between neutral silver clusters and DNA is rather weak (Δ*G* is equal to −1 to −9 kcal mol^−1^) as compared to charged particles (−55 to −63 kcal mol^−1^). This is in line with experimental fact that DNA-templated metal clusters are positively charged.^50–52^ The interaction between Au0 atoms and nucleobases is also rather weak (−4 to −6 kcal mol^−1^); however, the binding energy between diatomic cluster and DNA is higher (−25 to −26 kcal mol^−1^). This obstacle explains the existence of neutral gold nanoclusters stabilized by poly-cytosine and poly-adenine.^54^

Speaking about protein templates, the interaction of neutral metal atoms and diatomic clusters with amino acid residues is high for both silver and gold. This is in agreement with the fact that a large part of protein-templated nanoclusters consists of mostly reduced metal atoms.^15–17,22^

Moreover, the significant difference in the binding energy of neutral gold and silver atoms with nucleobases and amino acids apparently means that unlike DNA template neutral metal atoms are strongly bound to amino acid residues and can't freely diffuse in a polypeptide globula. This fact allows to make a conclusion that formation of metal nanoclusters in proteins occurs through the nucleation of Au atoms located on the neighboring amino acid residues, and the flexibility of the amino acid residue side chains and protein chain as a whole plays a significant role in this process.

Finally, based on the AIM analysis, we have found that for all complexes amino acid–Au_2_ bonds are partially electrostatic and partially covalent, the same is true for amino acid complexes with Au^+^.

## Conflicts of interest

There are no conflicts to declare.

## Supplementary Material

RA-010-D0RA06486F-s001
