# Supplementary material for: Comparative study of gold and silver interactions with amino acids and nucleobases
Source: RSC Adv. 2020 Sep 15;10(56):34149–60. doi: 10.1039/d0ra06486f (PMC9056802; doi:10.1039/d0ra06486f)
Supplement: RA-010-D0RA06486F-s001 [file RA-010-D0RA06486F-s001.pdf]

Electronic Supplementary Material (ESI) for RSC Advances.
This journal is © The Royal Society of Chemistry 2020

Comparative Study of Gold and Silver Interactions with Amino Acids and Nucleobases

Andrey A. Buglak[a,*], Alexei I. Kononov[a]

[a] St. Petersburg State University, 199034 Saint-Petersburg, Russia

*** Corresponding author:**

E-mail: andreybuglak@gmail.com

Supplementary information

Au⁺-Ala

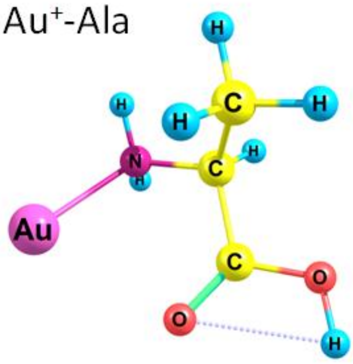

Au⁺-Arg

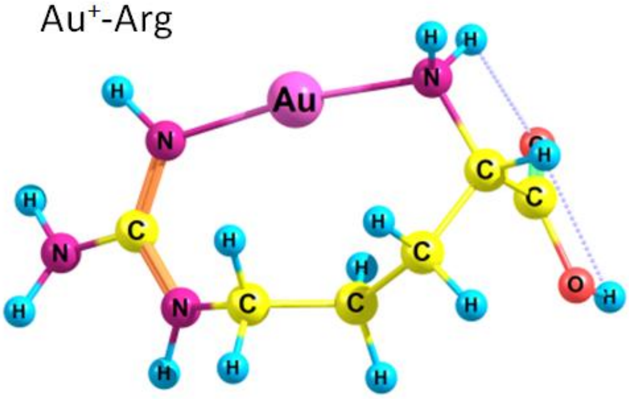

Au⁺-Asn

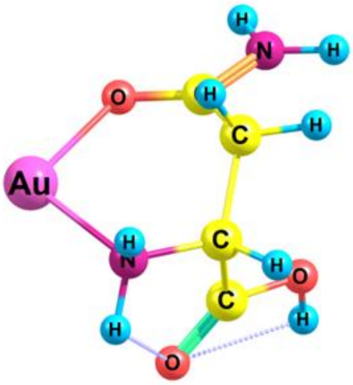

Au⁺-Asp

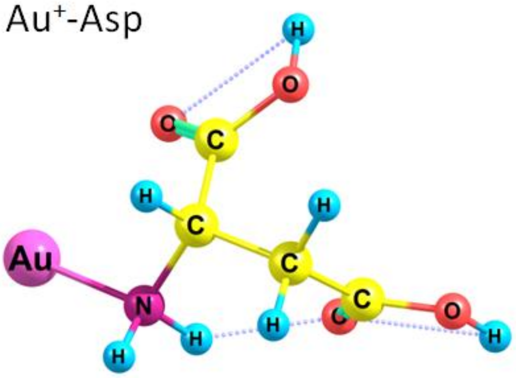

Au⁺-Asp(-H⁺)

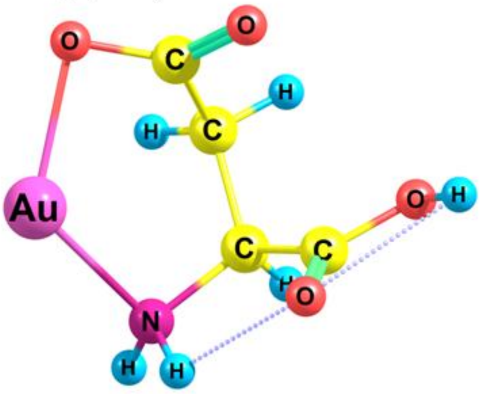

Au⁺-Cys

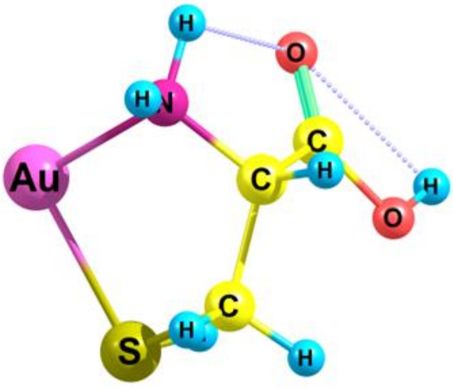

Au⁺-Cys(-H⁺)

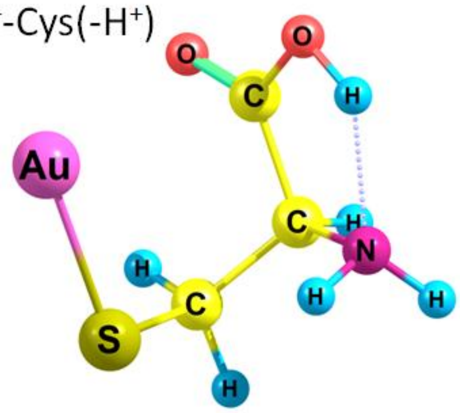

Au⁺-Gln

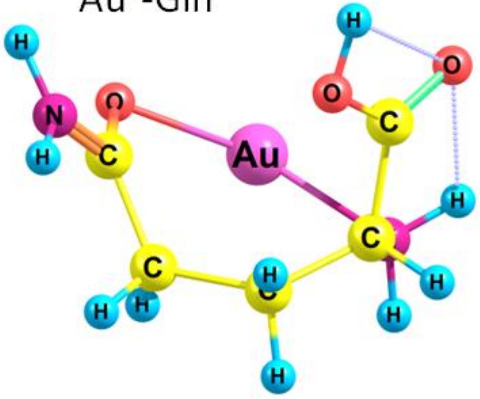

Au$^+$-Glu

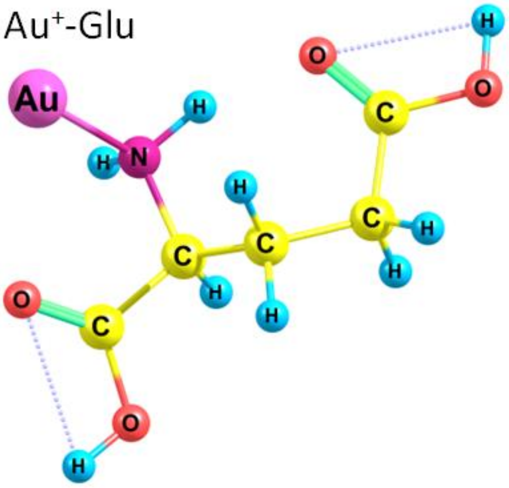

Au$^+$-Glu(-H$^+$)

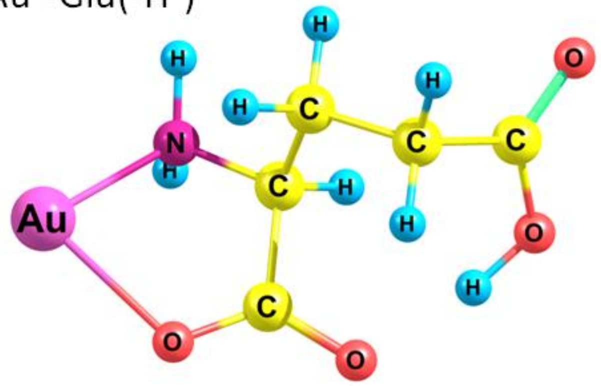

Au$^+$-Gly

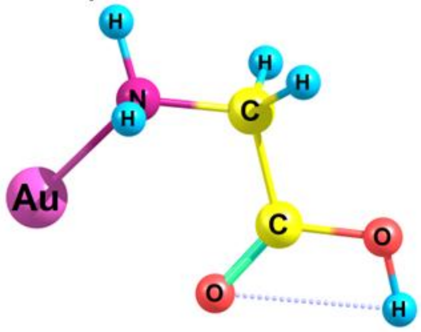

Au$^+$-His

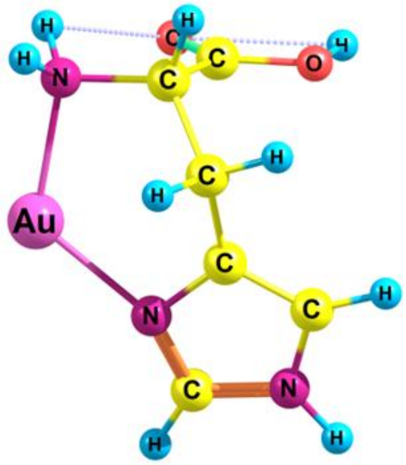

Au$^+$-Ile

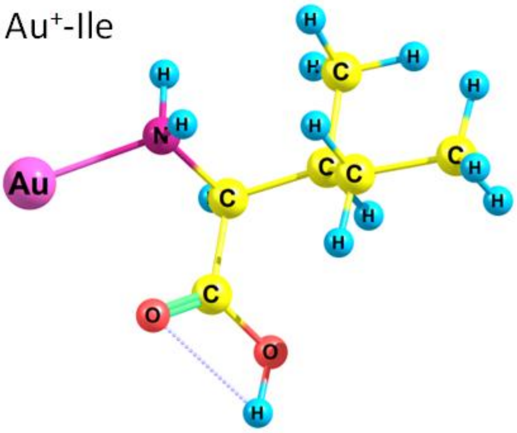

Au$^+$-Leu

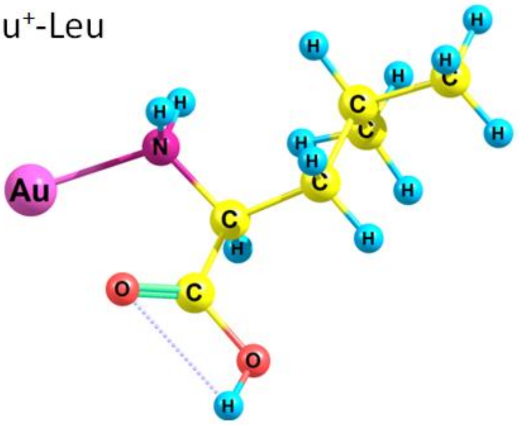

Au$^+$-Lys

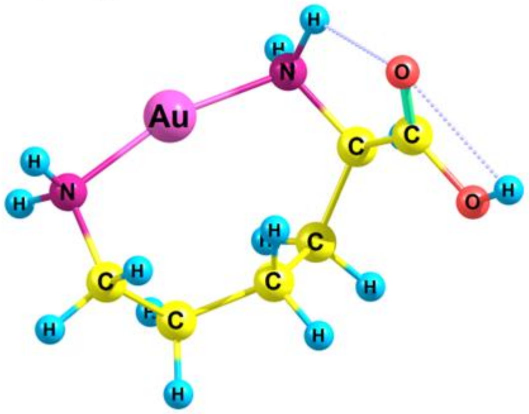

Au$^+$-Met

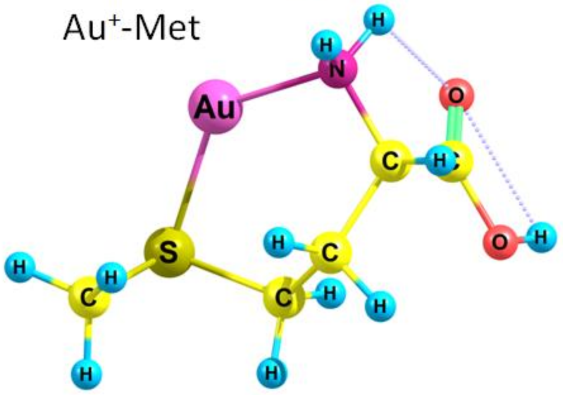

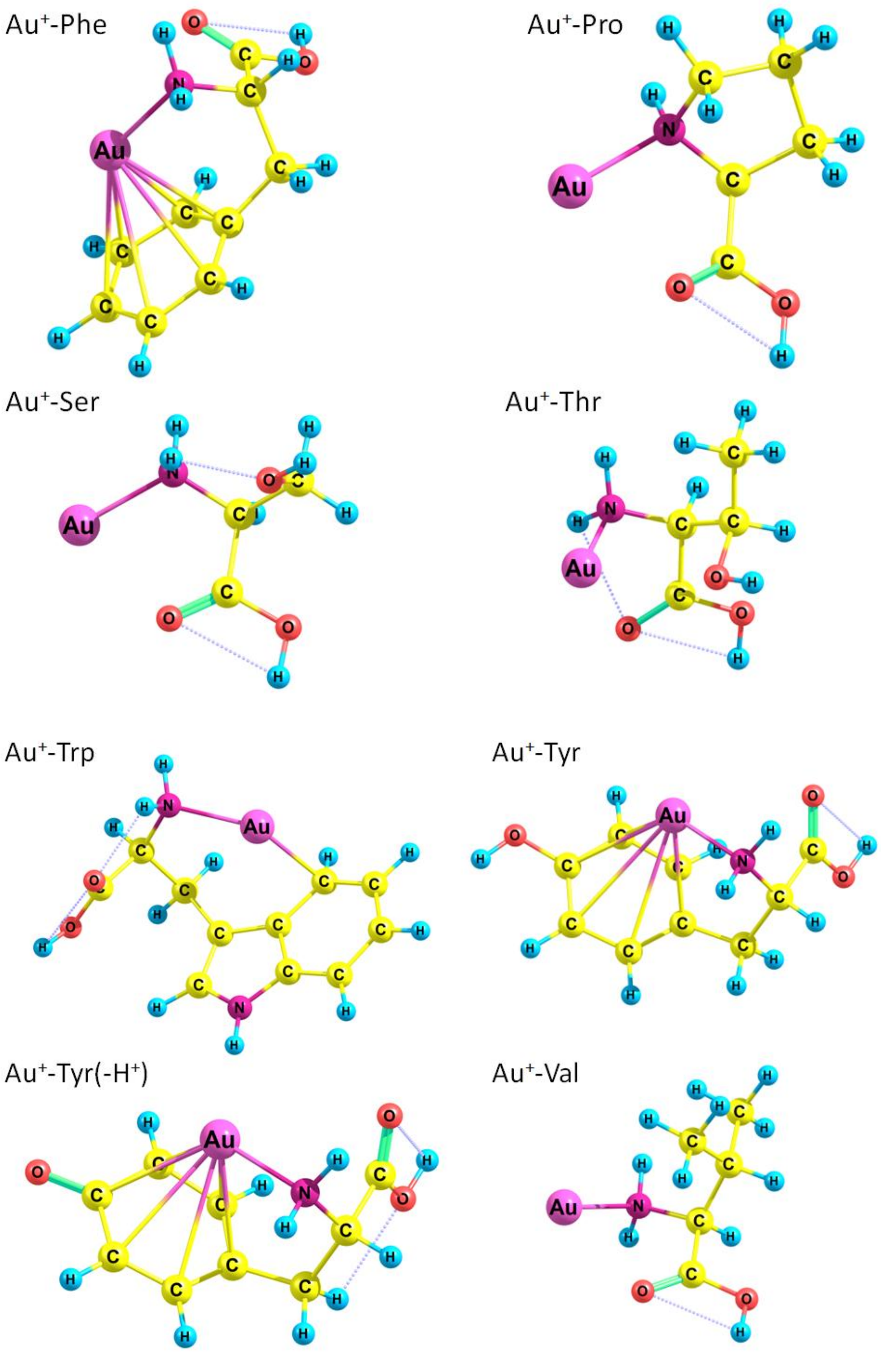

Figure S1. Geometry of amino acid complexes with Au^+ calculated at RI-MP2/def2-TZVP level of theory.

Interaction of Au_2 with disulfide bond was studied on dimethyldisulfide as a model compound instead of cysteine-cysteine S-S bond due to computational costs.

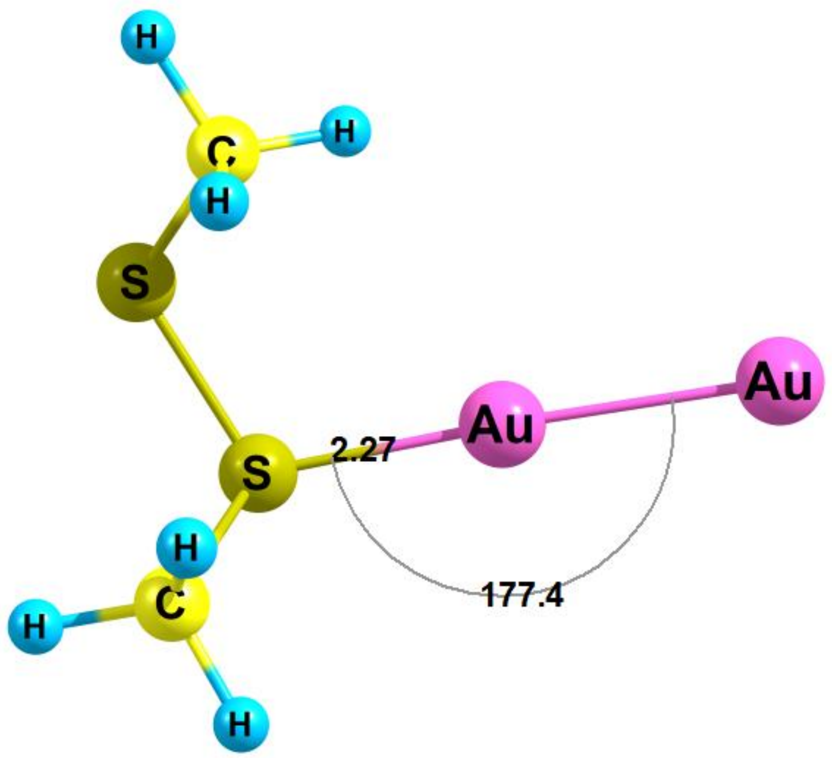

Figure S2. Anchoring of dimethyldisulfide with Au_2 cluster optimized with RI-MP2/def2-TZVP method.

Table S1. Gibbs free energies (in hartree) for Au^+ binding with amino acids (AA) calculated using PBE-D3/def2-TZVP and RI-MP2/ def2-TZVP method.

	PBE-D3			RI-MP2		
**AA**	**G(AA)**	**G(Complex)**	**ΔG**	**G(AA)**	**G(Complex)**	**ΔG**
Ala	-323.443	-459.159	-0.12	-323.089	-458.144	-0.10
Arg	-605.920	-741.737	-0.22	-605.218	-740.380	-0.21
Asn	-492.025	-627.750	-0.13	-491.495	-626.565	-0.12
Asp	-511.903	-647.623	-0.13	-511.380	-646.438	-0.10
Cys	-721.488	-857.223	-0.14	-720.799	-855.872	-0.12
Gln	-531.271	-667.010	-0.15	-530.688	-665.767	-0.13
Glu	-551.151	-686.881	-0.14	-550.57	-685.634	-0.11
Gly	-284.193	-419.903	-0.12	-283.895	-418.945	-0.10
His	-548.262	-684.019	-0.16	-547.644	-682.746	-0.15
Ile	-441.186	-576.903	-0.12	-440.661	-575.717	-0.10
Leu	-441.186	-576.904	-0.12	-440.662	-575.717	-0.10
Lys	-496.480	-632.273	-0.20	-495.892	-631.032	-0.19
Met	-799.977	-935.742	-0.17	-799.174	-934.279	-0.15
Phe	-554.219	-689.958	-0.15	-553.56	-688.645	-0.13
Pro	-400.748	-536.473	-0.13	-400.288	-535.351	-0.11
Ser	-398.620	-534.334	-0.12	-398.203	-533.260	-0.10
Thr	-437.873	-573.589	-0.12	-437.401	-572.459	-0.10
Trp	-685.671	-821.432	-0.17	-684.854	-819.961	-0.15
Tyr	-629.403	-765.146	-0.15	-628.683	-763.770	-0.13
Val	-401.939	-537.656	-0.12	-401.472	-536.527	-0.10
**Deprotonated amino acids:**						
Asp(-H^+)	-511.367	-647.252	-0.29	-510.843	-646.068	-0.27
Cys(-H^+)	-720.959	-856.883	-0.33	-720.270	-855.533	-0.31
Glu(-H^+)	-550.646	-686.506	-0.27	-550.063	-685.256	-0.24
Tyr(-H^+)	-628.865	-764.753	-0.29	-628.139	-763.375	-0.28

Au_2-Ala

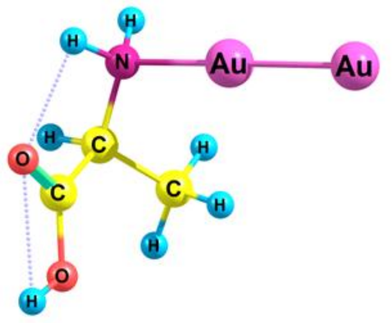

Au_2-Arg

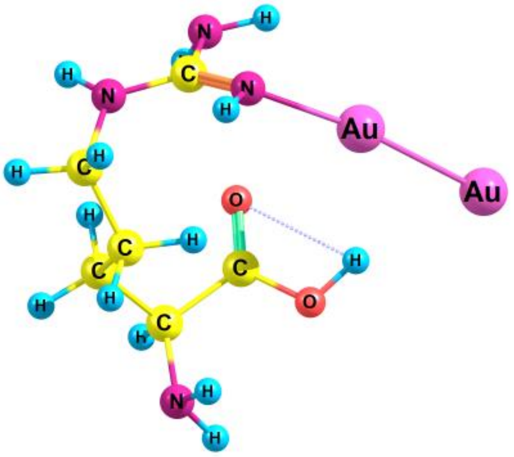

Au_2-Arg($+H^+$)

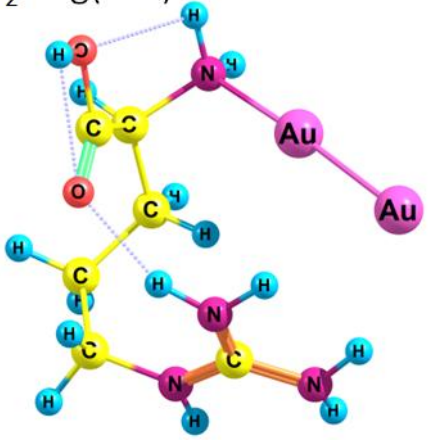

Au_2-Asn

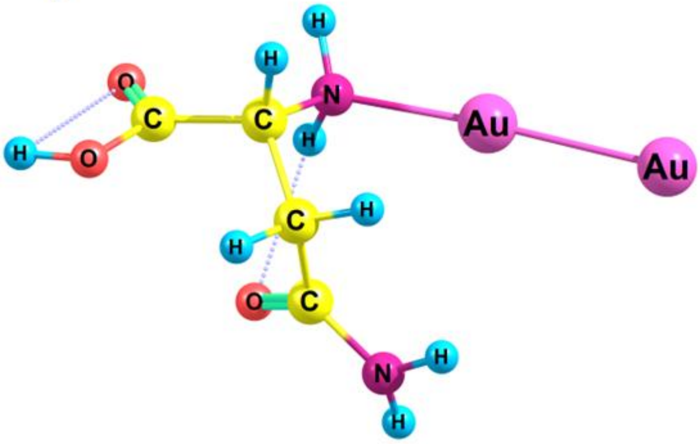

Au_2-Asp

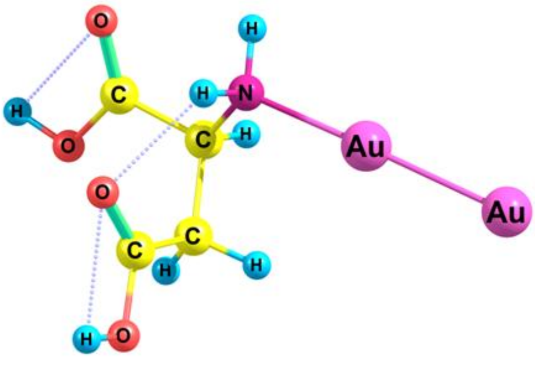

Au_2-Asp($-H^+$)

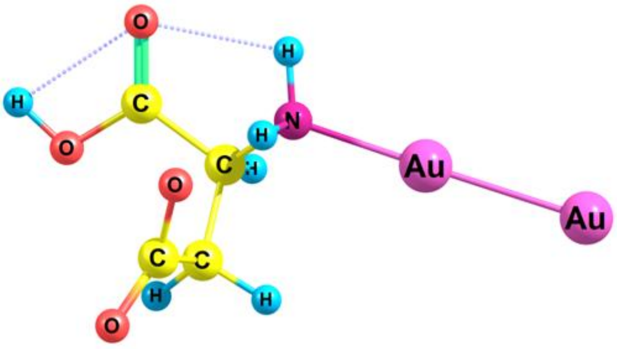

Au_2-Cys

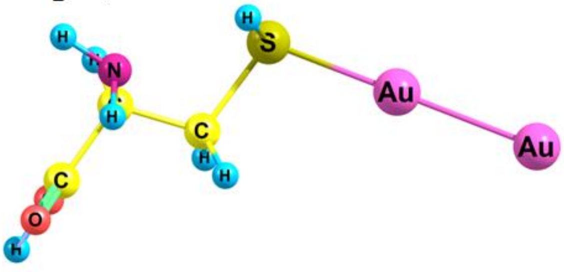

Au_2-Cys($-H^+$)

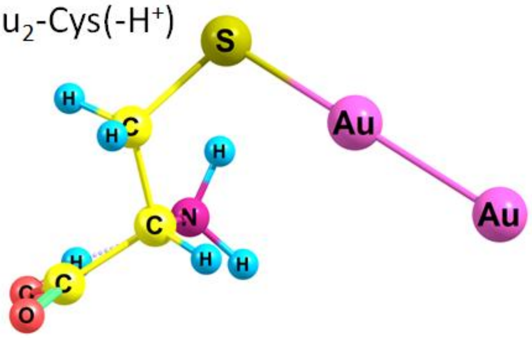

Au_2-Gln

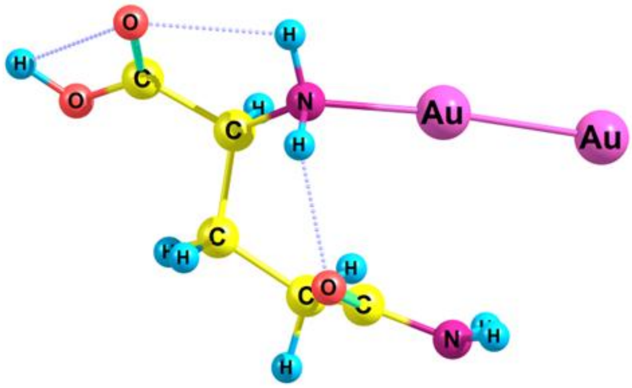

Au_2-Glu

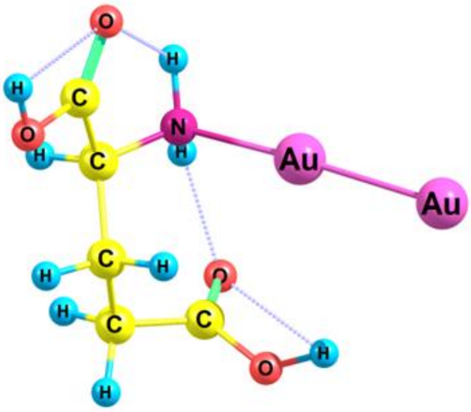

Au_2-Glu(-H^+)

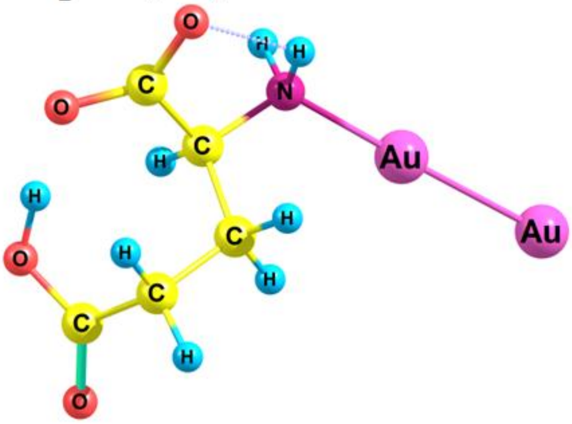

Au_2-Gly

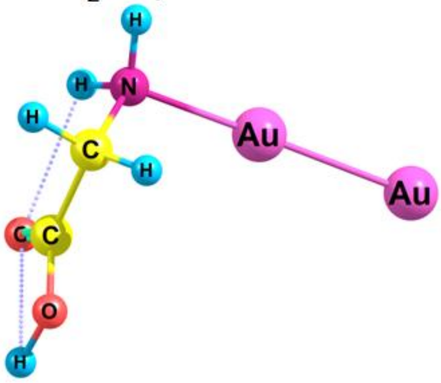

Au_2-His

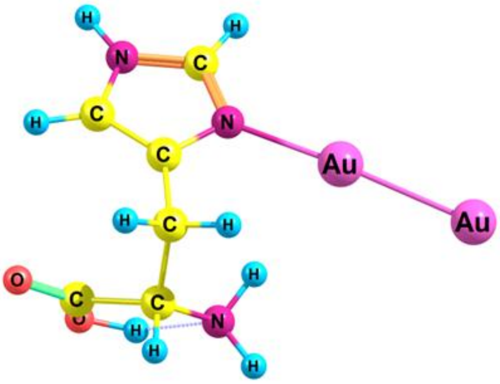

Au_2-His(+H^+)

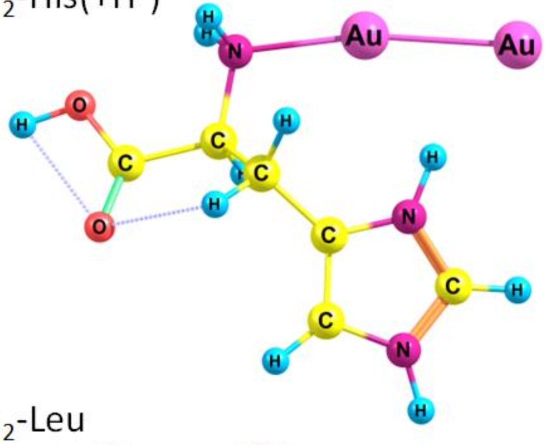

Au_2-Ile

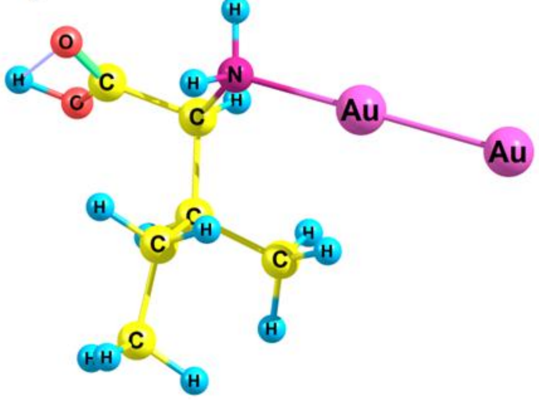

Au_2-Leu

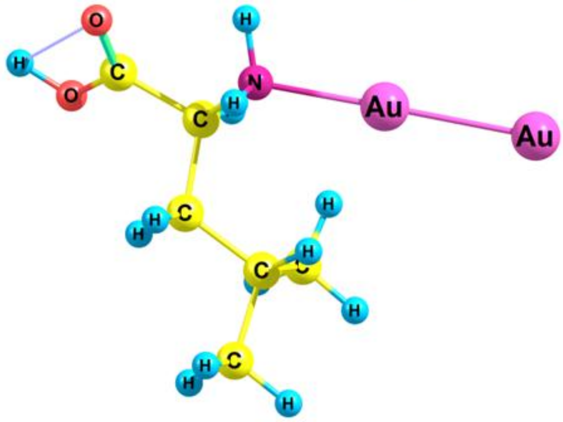

Au_2-Lys

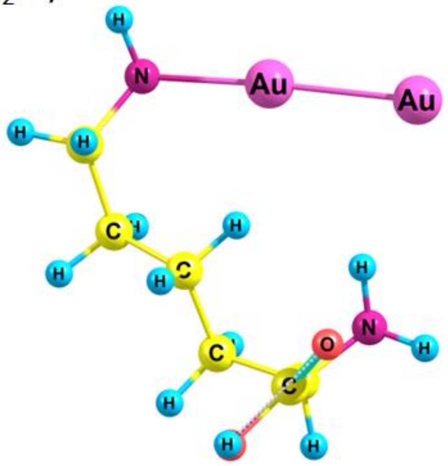

Au_2-Lys(+H^+)

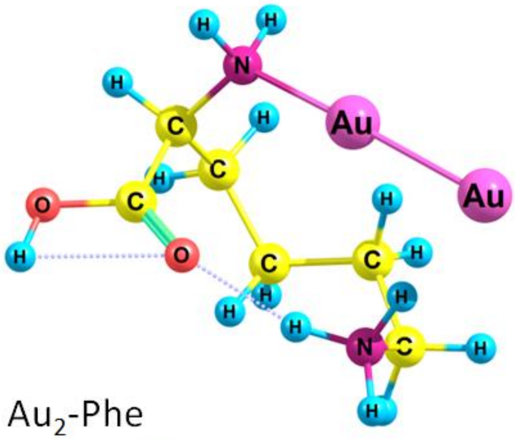

Au_2-Met

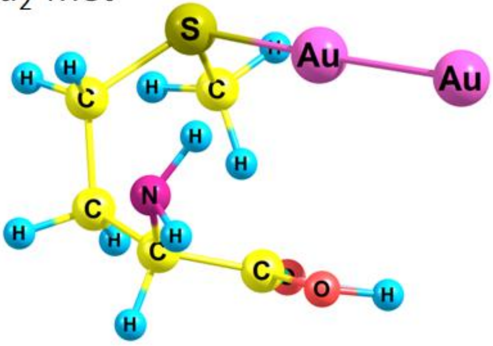

Au_2-Phe

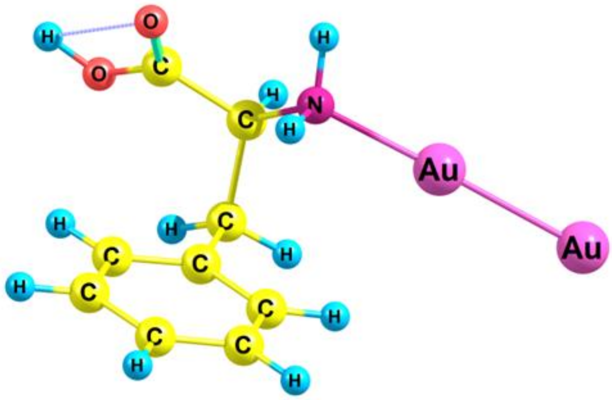

Au_2-Pro

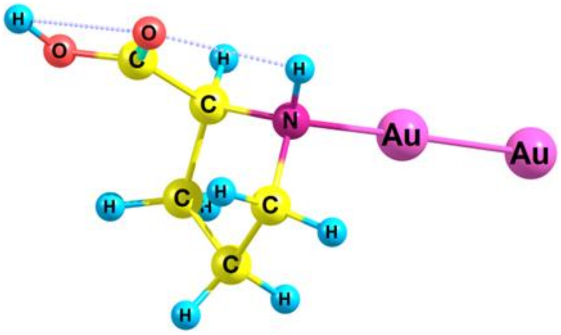

Au_2-Ser

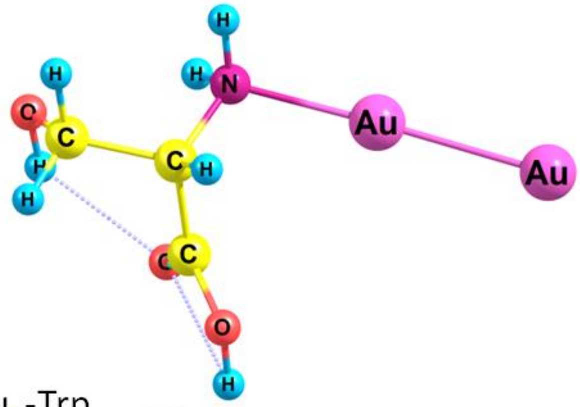

Au_2-Thr

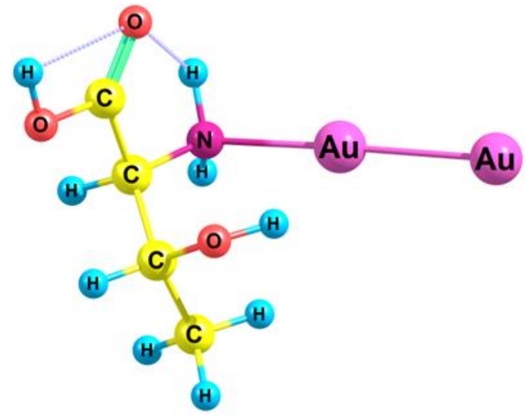

Au_2-Trp

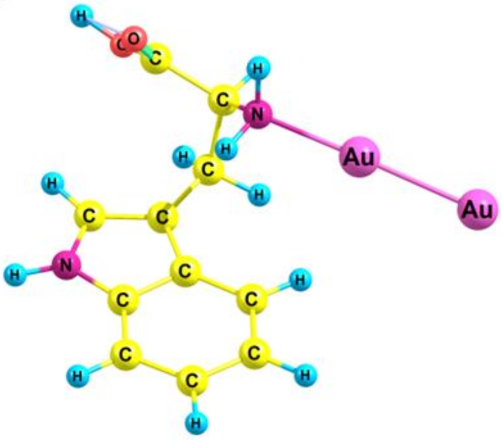

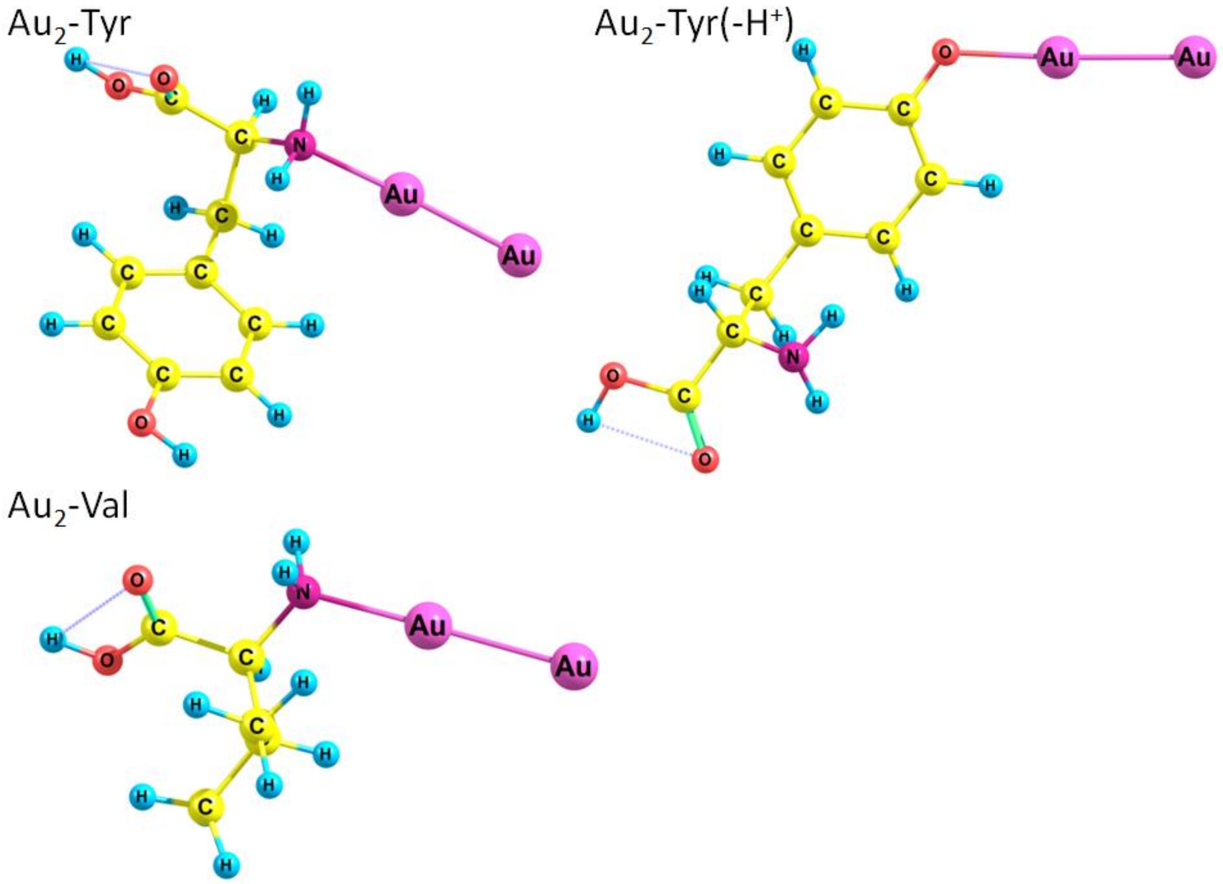

Figure S3. Geometry of amino acid complexes with Au_2 cluster calculated at RI-MP2/def2-TZVP level of theory.

Table S2. Gibbs free energies (in hartree) for amino acids (AA) and Au_2-AA complexes calculated using PBE-D3/def2-TZVP and RI-MP2/ def2-TZVP method.

	PBE-D3			RI-MP2		
**AA**	**G(AA)**	**G(Complex)**	**ΔG**	**G(AA)**	**G(Complex)**	**ΔG**
Ala	-323.443	-595.434	-0.03	-323.089	-593.750	-0.03
Arg	-605.920	-877.931	-0.05	-605.218	-875.900	-0.05
Asn	-492.025	-764.021	-0.03	-491.495	-762.166	-0.04
Asp	-511.903	-783.899	-0.03	-511.380	-782.043	-0.04
Cys	-721.488	-993.479	-0.03	-720.799	-991.457	-0.03
Gln	-531.271	-803.270	-0.04	-530.688	-801.361	-0.05
Glu	-551.151	-823.143	-0.03	-550.57	-821.233	-0.04
Gly	-284.193	-556.182	-0.03	-283.895	-554.553	-0.03
His	-548.262	-820.267	-0.04	-547.644	-818.317	-0.05
Ile	-441.186	-713.177	-0.03	-440.661	-711.322	-0.03
Leu	-441.186	-713.181	-0.03	-440.662	-711.325	-0.04
Lys	-496.480	-768.474	-0.03	-495.892	-766.556	-0.04
Met	-799.977	-1071.978	-0.04	-799.174	-1069.847	-0.04
Phe	-554.219	-826.215	-0.03	-553.56	-824.224	-0.04
Pro	-400.748	-672.745	-0.03	-400.288	-670.957	-0.04
Ser	-398.620	-670.606	-0.02	-398.203	-668.861	-0.03
Thr	-437.873	-709.863	-0.03	-437.401	-708.062	-0.03
Trp	-685.671	-957.666	-0.03	-684.854	-955.523	-0.04
Tyr	-629.403	-901.399	-0.03	-628.683	-899.347	-0.04
Val	-401.939	-673.930	-0.03	-401.472	-672.133	-0.03
**Deprotonated amino acids:**						
Asp($-H^+$)	-511.367	-783.394	-0.06	-510.843	-781.537	-0.07
Cys($-H^+$)	-720.959	-993.002	-0.08	-720.270	-990.983	-0.09
Glu($-H^+$)	-550.646	-822.662	-0.05	-550.063	-820.747	-0.06
Tyr ($-H^+$)	-628.865	-900.886	-0.06	-628.139	-898.829	-0.06
**Amino acids with protonated side chain:**						
Arg($+H^+$)	-606.328	-878.317	-0.03	-605.620	-876.283	-0.04
His($+H^+$)	-548.635	-820.624	-0.03	-548.009	-818.673	-0.04
Lys($+H^+$)	-496.860	-768.850	-0.03	-496.267	-766.930	-0.04

Cartesian coordinates of most favorable conformations of free amino acids (AA), AA complexes with Au^+, and AA complexes with Au_2 optimized using RI-MP2/def2-TZVP method:

Alanine

N	-1.055131000	1.346210000	0.083963000
C	-0.069766000	0.331147000	0.399952000
C	0.936618000	0.098510000	-0.712204000
O	0.785845000	0.411550000	-1.870610000
H	-0.593779000	2.234893000	-0.085291000
H	-1.508023000	1.101641000	-0.792932000
H	0.486245000	0.644263000	1.287554000
C	-0.769727000	-0.992888000	0.695945000
H	-1.481102000	-0.847506000	1.507993000
H	-0.054551000	-1.766406000	0.974607000
H	-1.319264000	-1.329123000	-0.186900000
O	2.038731000	-0.545007000	-0.261422000
H	2.603905000	-0.687284000	-1.040655000

Au^+-Alanine

C	-1.329476000	0.561933000	0.115230000
C	0.065965000	0.553765000	0.721657000
C	0.772554000	-0.735594000	0.338944000
N	0.846828000	1.716908000	0.223620000
O	1.540716000	-0.851169000	-0.593976000
O	0.393543000	-1.721594000	1.137865000
Au	1.516252000	1.484805000	-1.735372000
H	-1.280060000	0.470277000	-0.971993000
H	-1.853984000	1.483927000	0.371518000
H	-1.907195000	-0.273977000	0.507785000
H	0.005884000	0.605410000	1.811754000
H	1.668848000	1.866752000	0.812105000
H	0.273975000	2.557906000	0.308607000
H	0.816150000	-2.548346000	0.835255000

Au_2-Alanine

N	-0.697568000	1.225316000	0.102013000
C	0.083474000	0.159498000	0.763753000
C	1.457887000	0.186060000	0.126517000
O	1.937882000	1.171880000	-0.382103000
H	-1.492938000	1.475416000	0.686352000
H	-0.097550000	2.047997000	0.017889000
H	0.251188000	0.423647000	1.817653000
C	-0.619227000	-1.182087000	0.687761000
H	-1.637704000	-1.087446000	1.068548000
H	-0.089951000	-1.919852000	1.289528000
H	-0.672922000	-1.531351000	-0.343157000
O	2.098917000	-0.985844000	0.248890000
H	2.977623000	-0.858158000	-0.151585000
Au	-2.134297000	0.141788000	-4.097467000

Au	-1.364813000	0.733135000	-1.834593000

Arginine

N	-2.675024000	-0.187187000	1.269335000
C	-2.152603000	-0.163213000	-0.084988000
C	-1.010345000	-1.111134000	-0.398167000
O	-0.650100000	-1.355166000	-1.537171000
H	-1.913771000	-0.075755000	1.932153000
H	-3.076615000	-1.098501000	1.466952000
H	-2.962139000	-0.421196000	-0.772926000
C	-1.683223000	1.254143000	-0.454028000
H	-2.548200000	1.902142000	-0.300337000
H	-1.456142000	1.267497000	-1.524541000
C	-0.479686000	1.794830000	0.344253000
H	-0.270635000	1.175833000	1.222602000
H	-0.723090000	2.790177000	0.726046000
C	0.806549000	1.915359000	-0.479051000
H	0.595864000	2.429125000	-1.420535000
N	1.439385000	0.649712000	-0.804130000
H	1.536962000	2.533751000	0.048091000
C	2.119498000	-0.049954000	0.167771000
H	0.919565000	0.067952000	-1.451493000
N	2.581951000	0.358227000	1.289224000
N	2.269516000	-1.417379000	-0.147412000
H	2.343911000	1.332779000	1.455002000
H	2.415817000	-1.593732000	-1.134973000
H	3.018262000	-1.809346000	0.412872000
O	-0.384229000	-1.563521000	0.689043000
H	0.506783000	-1.891594000	0.392357000

Au^+-Arginine

N	1.665238000	0.173842000	1.645231000
C	2.393678000	-0.492745000	0.523941000
Au	-0.235671000	0.687307000	1.160241000
C	1.517629000	-1.424093000	-0.321151000
C	3.033910000	0.611969000	-0.294958000
N	-2.045558000	1.057861000	0.446658000
C	0.574431000	-0.716044000	-1.316261000
O	3.053972000	1.771014000	0.045874000
C	-2.463700000	0.774469000	-0.767894000
O	3.586139000	0.125294000	-1.409719000
C	-0.896009000	-1.138657000	-1.154670000
N	-1.852313000	-0.122111000	-1.568298000
N	-3.538475000	1.400431000	-1.308619000
H	2.192697000	1.021057000	1.880640000
H	1.681966000	-0.435211000	2.462971000
H	3.217069000	-1.078362000	0.948706000
H	2.191882000	-2.094980000	-0.855612000
H	0.947771000	-2.052834000	0.370985000
H	-2.709598000	1.609103000	0.977296000
H	0.634446000	0.370447000	-1.203134000
H	0.901168000	-0.922843000	-2.337960000

H	4.027648000	0.866154000	-1.865491000
H	-1.101322000	-2.031931000	-1.749092000
H	-1.120705000	-1.399496000	-0.119339000
H	-2.107908000	-0.085830000	-2.543822000
H	-4.112347000	0.918538000	-1.981496000
H	-3.965041000	2.165652000	-0.813023000

Au_2-Arginine

N	3.104079000	-2.462519000	-0.032750000
C	3.086072000	-1.178758000	0.645892000
C	1.791933000	-0.794957000	1.368127000
O	1.653152000	0.278664000	1.924934000
H	2.292042000	-2.536644000	-0.639096000
H	2.994342000	-3.208640000	0.647221000
H	3.863552000	-1.190853000	1.416776000
C	3.410817000	-0.050742000	-0.333742000
H	4.405278000	-0.228125000	-0.750607000
H	3.442909000	0.879913000	0.236911000
C	2.377239000	0.059320000	-1.454187000
H	1.385163000	-0.218910000	-1.081725000
H	2.613974000	-0.636156000	-2.264135000
C	2.289114000	1.471311000	-2.030275000
H	3.265899000	1.802913000	-2.388406000
N	1.853869000	2.449166000	-1.040912000
H	1.620397000	1.496314000	-2.895936000
C	0.696712000	2.317281000	-0.350929000
H	2.545780000	3.054059000	-0.626050000
N	-0.355750000	1.701398000	-0.823299000
N	0.662806000	2.922529000	0.875903000
H	-0.269311000	1.465338000	-1.806461000
H	1.431796000	2.648564000	1.477283000
H	-0.227345000	2.776829000	1.338292000
O	0.854577000	-1.737274000	1.295182000
H	-0.006244000	-1.379995000	1.647968000
Au	-1.421646000	0.477109000	0.412422000
Au	-2.348876000	-1.129319000	2.046864000

Arginine(+H^+)

N	-0.634712000	-1.057377000	1.610030000
C	-1.617130000	-0.298442000	0.836572000
C	-1.553930000	-0.762779000	-0.600803000
O	-0.740364000	-1.548225000	-1.044084000
H	-0.724788000	-0.822237000	2.595401000
H	-0.815154000	-2.055355000	1.527628000
H	-2.644866000	-0.481258000	1.173093000
C	-1.375154000	1.218158000	0.936224000
H	-1.261538000	1.465119000	1.997719000
H	-2.294109000	1.707269000	0.606581000
C	-0.220258000	1.828894000	0.135377000
H	-0.331505000	2.915560000	0.197785000
H	-0.316112000	1.576081000	-0.925760000
C	1.200114000	1.502938000	0.592239000

H	1.899960000	2.135107000	0.045670000
N	1.520101000	0.102822000	0.352158000
H	1.324084000	1.716728000	1.657246000
C	2.367471000	-0.367387000	-0.540766000
H	0.848332000	-0.559215000	0.800828000
N	2.196779000	-1.611127000	-0.997547000
H	2.928751000	-2.090586000	-1.494103000
H	1.269054000	-2.015554000	-0.957404000
O	-2.513605000	-0.194888000	-1.332646000
H	-2.440714000	-0.541349000	-2.240999000
N	3.390361000	0.369160000	-0.999081000
H	3.941543000	0.047063000	-1.777062000
H	3.741729000	1.144974000	-0.463537000

Au_2-Arginine($+H^+$)

N	-2.136391000	0.463709000	1.612437000
C	-1.773879000	0.940970000	0.246891000
C	-2.145138000	-0.183910000	-0.701167000
O	-1.400987000	-0.736320000	-1.480554000
H	-1.983984000	1.219754000	2.281692000
H	-3.133600000	0.243353000	1.640793000
H	-2.394933000	1.804582000	-0.023509000
C	-0.302264000	1.321623000	0.243037000
H	0.243205000	0.486143000	0.686193000
H	-0.198339000	2.143735000	0.957915000
C	0.326738000	1.741026000	-1.094117000
H	0.871392000	2.675376000	-0.941488000
H	-0.435291000	1.956741000	-1.848472000
C	1.302291000	0.720246000	-1.702224000
H	0.773738000	-0.093543000	-2.191276000
N	2.224975000	0.161538000	-0.720244000
H	1.901857000	1.204657000	-2.473963000
C	2.016373000	-0.964963000	-0.033582000
H	3.012065000	0.728721000	-0.438752000
N	2.822331000	-1.292080000	0.985421000
H	2.466239000	-1.992385000	1.632380000
H	3.459952000	-0.609075000	1.362630000
O	-3.440767000	-0.481984000	-0.570159000
H	-3.650119000	-1.215074000	-1.179141000
N	1.056867000	-1.816448000	-0.369699000
H	0.801935000	-2.507624000	0.340756000
H	0.311375000	-1.523278000	-0.990157000
Au	0.382200000	-3.214303000	2.629138000
Au	-0.999022000	-1.224227000	2.143749000

Asparagine

N	1.566039000	1.701867000	0.016565000
C	0.598988000	0.609585000	0.157172000
C	1.357021000	-0.713531000	-0.011517000
O	0.827145000	-1.759434000	-0.315169000
H	1.246836000	2.528108000	0.510038000
H	1.671304000	1.957634000	-0.961690000

H	0.266636000	0.619453000	1.201911000
C	-0.632040000	0.689057000	-0.759109000
H	-0.440871000	0.150328000	-1.690744000
H	-0.830027000	1.736097000	-0.986913000
C	-1.895252000	0.173237000	-0.085019000
O	-2.747007000	0.945009000	0.336629000
N	-1.982320000	-1.172465000	0.015869000
H	-1.172660000	-1.741311000	-0.195310000
H	-2.753158000	-1.553640000	0.541027000
O	2.661686000	-0.615687000	0.233902000
H	2.805789000	0.352563000	0.373087000

Au^{+}-Asparagine

N	0.880271000	0.007314000	-1.689704000
C	1.058394000	-0.636728000	-0.361839000
Au	-0.832939000	1.213842000	-1.502807000
C	1.681331000	0.408597000	0.549539000
C	-0.225739000	-1.269678000	0.209212000
O	-1.663465000	0.691058000	0.514178000
O	1.671511000	-0.005011000	1.820674000
O	2.145396000	1.446270000	0.148061000
C	-1.170771000	-0.356927000	0.980365000
N	-1.506523000	-0.765226000	2.201503000
H	1.701549000	0.589509000	-1.872189000
H	0.819228000	-0.696234000	-2.424411000
H	1.800616000	-1.439441000	-0.449995000
H	0.076895000	-2.097064000	0.851854000
H	-0.800372000	-1.706959000	-0.614130000
H	2.131057000	0.669562000	2.356463000
H	-1.093383000	-1.576906000	2.630402000
H	-2.168056000	-0.212973000	2.730824000

Au_2-Asparagine

N	-0.105114000	0.561056000	-1.025906000
C	0.692919000	-0.463681000	-0.324585000
Au	-2.050572000	-0.058571000	-1.479300000
C	1.955302000	0.180619000	0.224902000
C	-0.146304000	-1.171241000	0.733484000
O	-0.301703000	0.931278000	1.840088000
O	2.607193000	-0.636916000	1.070785000
O	2.358420000	1.267336000	-0.111601000
C	-0.850519000	-0.136465000	1.584150000
N	-2.065203000	-0.488873000	2.064165000
H	-0.156513000	1.370711000	-0.401634000
H	0.408832000	0.879004000	-1.846474000
H	1.012152000	-1.205170000	-1.063209000
H	0.503813000	-1.763603000	1.381851000
H	-0.855627000	-1.845280000	0.250477000
H	3.407139000	-0.157141000	1.349683000
H	-2.595598000	-1.201395000	1.586533000
H	-2.588987000	0.233530000	2.537440000
Au	-4.338915000	-0.877223000	-1.878351000

Aspartic acid

N	0.796932000	1.838360000	-0.198092000
C	0.678667000	0.688682000	0.679640000
C	1.335569000	-0.604207000	0.209347000
O	1.040189000	-1.707683000	0.606816000
H	1.773844000	2.087903000	-0.307738000
H	0.458142000	1.581236000	-1.120682000
H	1.185549000	0.933635000	1.620364000
C	-0.778993000	0.410225000	1.011253000
C	-1.544469000	-0.025821000	-0.205120000
H	-1.243741000	1.314841000	1.407886000
H	-0.870256000	-0.374775000	1.763630000
O	-1.081778000	-0.156388000	-1.317450000
O	-2.839457000	-0.264544000	0.080358000
H	-3.251212000	-0.544559000	-0.755711000
O	2.339806000	-0.382445000	-0.664263000
H	2.699769000	-1.261992000	-0.874000000

Au^+-Aspartic acid

N	1.562781000	-0.191095000	-1.551289000
C	1.753089000	0.123301000	-0.111161000
Au	2.913052000	-1.552346000	-2.330876000
C	2.010615000	-1.175985000	0.650500000
C	0.574073000	0.910535000	0.451858000
O	-0.795816000	-0.918037000	-0.280172000
O	2.465089000	-2.183776000	0.156800000
O	1.718077000	-1.033524000	1.938574000
C	-0.689043000	0.088888000	0.396957000
O	-1.653947000	0.598079000	1.148639000
H	1.569261000	0.674996000	-2.090347000
H	0.623870000	-0.606216000	-1.627495000
H	2.666330000	0.718742000	-0.016869000
H	0.417775000	1.830132000	-0.121109000
H	0.771708000	1.213095000	1.480214000
H	1.939458000	-1.869055000	2.393096000
H	-2.447253000	0.038774000	1.046530000

Au_2- Aspartic acid

N	1.645134000	-0.425945000	-1.450390000
C	1.551038000	0.339937000	-0.196217000
Au	0.512262000	0.384274000	-3.014888000
C	2.434483000	-0.304340000	0.860662000
C	0.107448000	0.508682000	0.247276000
O	0.003420000	-1.892247000	0.397205000
O	3.327693000	-1.077166000	0.614645000
O	2.138035000	0.137084000	2.097747000
C	-0.539546000	-0.814774000	0.533585000
O	-1.800709000	-0.672195000	0.963095000
H	1.341135000	-1.379851000	-1.241502000
H	2.629745000	-0.499591000	-1.706243000
H	1.964081000	1.335860000	-0.384554000

H	-0.464466000	1.010898000	-0.538307000
H	0.049367000	1.128069000	1.143632000
H	2.770902000	-0.293205000	2.700360000
H	-2.149551000	-1.570495000	1.103449000
Au	-0.845311000	1.372830000	-4.814569000

Aspartic acid(-H$^+$)

C	0.476188000	2.944063000	-4.534653000
C	1.043077000	3.169235000	-3.136258000
C	2.018724000	2.099600000	-2.739801000
N	1.689206000	4.471085000	-3.087889000
O	3.162320000	2.248524000	-2.363184000
O	1.463971000	0.853211000	-2.798128000
C	-0.654400000	3.956147000	-4.901801000
H	0.061128000	1.938415000	-4.625355000
H	1.280081000	3.044467000	-5.270071000
H	0.195477000	3.078307000	-2.437661000
H	2.054843000	4.639675000	-2.155614000
H	0.918548000	5.120688000	-3.260843000
H	2.178431000	0.264673000	-2.503544000
O	-1.128633000	3.803974000	-6.046798000
O	-0.965899000	4.797103000	-4.013142000

Au$^+$-Aspartic acid(-H$^+$)

N	1.846161000	0.277541000	-1.492024000
C	1.701842000	0.513645000	-0.015449000
Au	-0.120589000	-0.216535000	-2.053048000
C	2.150620000	-0.758823000	0.677900000
C	0.281149000	0.911337000	0.441475000
O	-1.638076000	-0.209869000	-0.618272000
O	2.445701000	-1.774528000	0.090099000
O	2.216199000	-0.588852000	1.998740000
C	-0.768909000	-0.245751000	0.377515000
O	-0.724263000	-1.091260000	1.258797000
H	2.484220000	-0.511729000	-1.615278000
H	2.240829000	1.096438000	-1.946180000
H	2.394300000	1.307187000	0.284743000
H	-0.047962000	1.780824000	-0.132488000
H	0.365950000	1.201187000	1.491663000
H	2.387279000	-1.463719000	2.389958000

Au_2-Aspartic acid(-H$^+$)

N	1.383508000	-0.699768000	-1.351898000
C	1.414206000	0.276456000	-0.244600000
Au	0.532258000	-0.028643000	-3.094359000
C	2.467867000	-0.168889000	0.749199000
C	0.029151000	0.378712000	0.378860000
O	0.127661000	-2.003347000	0.521227000
O	3.394449000	-0.907100000	0.492995000
O	2.316363000	0.413822000	1.955744000
C	-0.445987000	-0.964761000	0.995138000
O	-1.325588000	-0.897648000	1.862969000

H	0.863592000	-1.491499000	-0.867506000
H	2.338122000	-1.025103000	-1.506998000
H	1.743514000	1.252413000	-0.619296000
H	-0.677791000	0.671131000	-0.404171000
H	0.010409000	1.147797000	1.150128000
H	3.032953000	0.052283000	2.504795000
Au	-0.446710000	0.809146000	-5.217819000

Cysteine

N	0.233915000	1.765316000	0.175762000
C	0.273903000	0.444722000	0.795364000
C	1.076197000	-0.507144000	-0.101683000
O	1.027150000	-1.709271000	0.006536000
H	-0.571281000	1.817314000	-0.446532000
H	0.146461000	2.499064000	0.869037000
H	0.854962000	0.523568000	1.722079000
C	-1.080778000	-0.171516000	1.127581000
S	-2.161619000	-0.311101000	-0.324095000
H	-1.471093000	-1.301058000	-0.905781000
H	-0.947957000	-1.153920000	1.578723000
H	-1.616068000	0.467230000	1.833461000
O	1.850204000	0.120559000	-0.989005000
H	1.629445000	1.075354000	-0.862054000

Au^+-Cysteine

N	-0.194444000	1.618881000	-0.927449000
C	0.001766000	0.160876000	-0.987253000
Au	0.417780000	2.051423000	1.171352000
C	1.323549000	-0.315476000	-0.364841000
C	-1.213033000	-0.471629000	-0.324712000
S	1.565774000	0.027478000	1.419594000
O	-2.214095000	0.144916000	-0.056003000
O	-1.018763000	-1.775703000	-0.101664000
H	0.330831000	2.096302000	-1.657313000
H	-1.188384000	1.817222000	-1.055905000
H	0.013820000	-0.189473000	-2.027744000
H	1.433720000	-1.389687000	-0.513616000
H	2.167322000	0.183249000	-0.848744000
H	0.601246000	-0.787383000	1.882386000
H	-1.852090000	-2.147996000	0.245910000

Au_2-Cysteine

Au	-0.902594000	10.352302000	4.919163000
Au	-0.122117000	11.659540000	2.973034000
C	1.880511000	13.976132000	1.835389000
C	2.751204000	14.476821000	0.680168000
C	3.737650000	15.477626000	1.260482000
N	3.394202000	13.351546000	0.033418000
O	4.907589000	15.252916000	1.457890000
O	3.132179000	16.641703000	1.568832000
S	0.564952000	12.897977000	1.198446000
H	1.380553000	14.799960000	2.345973000

H	2.487282000	13.426198000	2.557351000
H	2.111747000	14.998069000	-0.037164000
H	4.223461000	13.095835000	0.565811000
H	3.721211000	13.615010000	-0.890165000
H	3.812062000	17.211180000	1.971188000
H	1.413442000	12.093188000	0.532464000

Cysteine(-H$^+$)

N	1.393272000	1.683740000	0.070984000
C	0.393693000	0.639340000	0.322555000
C	0.982478000	-0.763181000	0.117590000
O	0.128216000	-1.765674000	0.121404000
H	2.301852000	1.247015000	0.225673000
H	1.373140000	1.875308000	-0.928868000
H	0.086347000	0.687245000	1.374486000
C	-0.863437000	0.885578000	-0.513887000
S	-2.272235000	-0.165946000	-0.038388000
H	-0.851078000	-1.362241000	0.125398000
H	-1.119072000	1.943242000	-0.393100000
H	-0.603395000	0.733531000	-1.571873000
O	2.189523000	-0.933539000	-0.003204000

Au$^+$-Cysteine(-H$^+$)

N	-2.495836000	0.836347000	1.018690000
C	-2.178015000	0.375035000	-0.328893000
C	-1.730267000	-1.093018000	-0.273598000
O	-1.287629000	-1.684033000	-1.231814000
H	-1.702771000	1.368202000	1.379375000
H	-3.314930000	1.432203000	1.029145000
H	-3.096121000	0.362345000	-0.927496000
C	-1.147857000	1.221397000	-1.076474000
S	0.315755000	1.663322000	-0.084491000
H	-0.851669000	0.710933000	-1.992224000
H	-1.589655000	2.188573000	-1.337949000
O	-1.923675000	-1.662301000	0.917912000
H	-2.287785000	-0.916029000	1.464555000
Au	1.115591000	-0.341884000	0.352487000

Au$_2$-Cysteine(-H$^+$)

N	-2.783535000	-0.233118000	1.441048000
C	-2.626465000	0.404543000	0.131893000
C	-3.867467000	1.240487000	-0.164931000
O	-3.946116000	2.046047000	-1.064841000
H	-2.329142000	-1.149770000	1.371491000
H	-2.258269000	0.285203000	2.139079000
H	-1.744195000	1.048228000	0.073268000
C	-2.509414000	-0.656093000	-0.972647000
S	-1.259961000	-1.932213000	-0.619536000
H	-3.470374000	-1.174299000	-1.078668000
H	-2.295961000	-0.140312000	-1.909488000
O	-4.896225000	0.950266000	0.648369000
H	-4.471894000	0.298661000	1.276605000

Au	2.492326000	0.630732000	0.841140000
Au	0.523427000	-0.709027000	0.038542000

Glutamine

N	-0.476523000	-1.470018000	0.529894000
C	-0.960842000	-0.095716000	0.525288000
C	-2.292778000	0.033691000	-0.188136000
O	-2.717246000	-0.753605000	-1.002360000
H	-1.019071000	-2.020271000	1.189738000
H	-0.676537000	-1.871233000	-0.386003000
H	-1.092864000	0.239421000	1.557556000
C	0.038377000	0.845111000	-0.158713000
H	-0.423037000	1.832039000	-0.228341000
H	0.199270000	0.498681000	-1.185486000
C	1.379818000	0.956115000	0.590850000
H	1.264508000	0.580214000	1.611553000
H	1.691722000	1.997966000	0.646892000
C	2.515844000	0.217115000	-0.093971000
O	3.417301000	0.807057000	-0.674365000
N	2.443929000	-1.134699000	-0.000928000
H	1.586223000	-1.555946000	0.340533000
H	3.110194000	-1.674320000	-0.530304000
O	-2.943140000	1.162465000	0.161914000
H	-3.753109000	1.189667000	-0.377464000

Au^+-Glutamine

C	0.738048000	1.799056000	-0.574439000
C	-0.778383000	1.798821000	-0.728094000
O	1.308199000	2.556745000	0.171987000
O	1.339223000	0.914734000	-1.379377000
C	-1.388043000	0.543430000	-1.377211000
N	-1.297694000	2.191343000	0.621830000
C	-1.548768000	-0.841512000	-0.687564000
Au	-0.788567000	0.514891000	1.679277000
C	-0.391777000	-1.548127000	0.001876000
O	-0.149904000	-1.426192000	1.238077000
N	0.330751000	-2.395104000	-0.715575000
H	-0.987761000	2.616643000	-1.432808000
H	2.301595000	1.043642000	-1.274081000
H	-0.822050000	0.411992000	-2.301497000
H	-2.402511000	0.806745000	-1.692262000
H	-0.781754000	3.022015000	0.924300000
H	-2.289293000	2.425232000	0.585046000
H	-1.910598000	-1.500752000	-1.483720000
H	-2.352714000	-0.805871000	0.050962000
H	0.128975000	-2.576665000	-1.685690000
H	1.078026000	-2.907066000	-0.265034000

Au_2-Glutatmine

N	-0.685586000	-1.464514000	0.314942000
C	-1.119633000	-0.067896000	0.519736000
C	-2.435499000	0.158875000	-0.197434000

O	-3.021940000	-0.683300000	-0.837002000
H	-1.516735000	-2.039288000	0.166947000
H	-0.135210000	-1.508613000	-0.549593000
H	-1.308564000	0.084362000	1.586955000
C	-0.071160000	0.939714000	0.046278000
H	-0.440178000	1.933099000	0.304145000
H	0.008437000	0.887903000	-1.043519000
C	1.314868000	0.739101000	0.644166000
H	1.269784000	0.575871000	1.725398000
H	1.902250000	1.650401000	0.486598000
C	2.082619000	-0.386448000	-0.028977000
O	1.757036000	-0.824896000	-1.129855000
N	3.198954000	-0.795200000	0.623939000
H	3.224598000	-0.700717000	1.629766000
H	3.631687000	-1.632031000	0.257066000
O	-2.887622000	1.412097000	-0.017008000
H	-3.737174000	1.468827000	-0.489540000
Au	0.492914000	-2.171463000	1.885424000
Au	1.975670000	-2.829214000	3.740895000

Glutamic acid

N	-0.784702000	-1.748151000	0.487121000
C	-0.800215000	-0.318325000	0.236641000
C	-2.198195000	0.230726000	0.034446000
O	-3.165917000	-0.430166000	-0.268251000
H	-1.148342000	-1.938310000	1.415528000
H	-1.429475000	-2.198431000	-0.158916000
H	-0.347167000	0.204379000	1.081417000
C	0.011781000	0.006962000	-1.024211000
C	1.448529000	-0.474303000	-0.925171000
H	-0.015827000	1.083901000	-1.200500000
H	-0.468238000	-0.484531000	-1.877367000
H	1.958951000	-0.383681000	-1.888365000
H	1.483683000	-1.529400000	-0.647281000
C	2.255829000	0.298894000	0.077795000
O	1.881367000	1.260375000	0.710361000
O	3.504522000	-0.208339000	0.197294000
H	3.955424000	0.352656000	0.851826000
O	-2.241272000	1.571021000	0.190671000
H	-3.158281000	1.834644000	-0.001520000

Au^{+}-Glutamic acid

N	-0.916895000	0.170343000	1.735691000
C	0.194566000	-0.438897000	0.968296000
Au	-0.404828000	1.859725000	2.791381000
C	0.481540000	0.351055000	-0.316810000
C	1.460619000	-0.485986000	1.809144000
O	-2.408809000	0.779166000	-0.436951000
O	1.708418000	0.249757000	2.739134000
O	2.279153000	-1.427101000	1.347561000
C	-0.383542000	0.002362000	-1.525350000
C	-1.819036000	0.444032000	-1.453045000

O	-2.398810000	0.418178000	-2.645272000
H	-1.305808000	-0.507969000	2.392236000
H	-1.653543000	0.422355000	1.043958000
H	-0.073070000	-1.469857000	0.715217000
H	1.516900000	0.152474000	-0.601185000
H	0.418163000	1.421119000	-0.092033000
H	3.101834000	-1.401391000	1.872313000
H	-0.386082000	-1.078326000	-1.704231000
H	0.049352000	0.444738000	-2.425231000
H	-3.331123000	0.683220000	-2.535824000

Au_2-Glutamic acid

N	-1.044894000	-0.255108000	1.540031000
C	0.217275000	-0.671360000	0.898904000
Au	-1.290854000	1.824639000	1.700628000
C	0.537189000	0.104734000	-0.372967000
C	1.290323000	-0.554070000	1.966434000
O	-2.761568000	-0.017317000	-0.780719000
O	1.055942000	-0.581922000	3.151272000
O	2.527045000	-0.479676000	1.448991000
C	-0.463774000	-0.126861000	-1.512056000
C	-1.791075000	0.534980000	-1.259795000
O	-1.766434000	1.828362000	-1.606215000
H	-1.061520000	-0.654795000	2.479857000
H	-1.840333000	-0.615685000	1.009590000
H	0.173389000	-1.743133000	0.650256000
H	1.519198000	-0.223879000	-0.716575000
H	0.607530000	1.171907000	-0.143244000
H	3.136331000	-0.450253000	2.208490000
H	-0.639310000	-1.194967000	-1.655330000
H	-0.042586000	0.286072000	-2.429886000
H	-2.583019000	2.236551000	-1.263010000
Au	-1.623468000	4.263357000	1.780094000

Glutamic acid($-H^+$)

N	1.965760000	1.604008000	0.780499000
C	0.922094000	0.626039000	0.450945000
C	1.489069000	-0.661873000	-0.190111000
O	2.595695000	-0.588716000	-0.747384000
H	2.439840000	1.268872000	1.615978000
H	2.663773000	1.476465000	0.044733000
H	0.391506000	0.358428000	1.368533000
C	-0.063534000	1.289844000	-0.515021000
C	-1.183203000	0.389546000	-1.050068000
H	0.517268000	1.670800000	-1.363099000
H	-0.498296000	2.157396000	-0.009669000
H	-0.750397000	-0.426236000	-1.633063000
H	-1.838066000	0.976956000	-1.695479000
C	-2.047559000	-0.182368000	0.063266000
O	-1.537563000	-1.203612000	0.721540000
O	-3.141099000	0.296395000	0.332121000
O	0.736913000	-1.697055000	-0.122377000

H	-0.549405000	-1.430980000	0.366106000

Au^+-Glutamic acid(-H^+)

N	2.079678000	1.113756000	0.891645000
C	0.843238000	0.423764000	0.446070000
C	1.033792000	-1.099469000	0.212394000
O	2.169920000	-1.547930000	-0.155007000
H	2.210109000	0.995066000	1.894435000
H	2.003339000	2.113424000	0.714877000
H	0.074144000	0.537551000	1.213633000
C	0.380945000	1.071147000	-0.858591000
C	-0.905684000	0.475156000	-1.435570000
H	1.185946000	0.976045000	-1.596348000
H	0.218208000	2.140989000	-0.682074000
H	-0.758384000	-0.575816000	-1.687971000
H	-1.166027000	1.021289000	-2.341835000
C	-2.061562000	0.647674000	-0.463515000
O	-2.193267000	-0.298827000	0.470167000
O	-2.797482000	1.608622000	-0.505716000
O	0.004949000	-1.781894000	0.336715000
H	-1.434588000	-0.947480000	0.412796000
Au	3.667270000	0.040241000	-0.156336000

Au_2-Glutamic acid(-H^+)

N	1.522439000	1.702872000	1.104186000
C	0.470968000	0.804226000	0.558342000
C	1.136207000	-0.561973000	0.244641000
O	2.370667000	-0.541463000	0.078027000
H	1.682337000	1.423132000	2.071845000
H	2.367295000	1.368058000	0.615034000
H	-0.307559000	0.682880000	1.313989000
C	-0.116980000	1.419356000	-0.709591000
C	-1.071697000	0.507279000	-1.487940000
H	0.718118000	1.695721000	-1.363063000
H	-0.632855000	2.346860000	-0.442680000
H	-0.537763000	-0.381222000	-1.830970000
H	-1.446401000	1.052462000	-2.354647000
C	-2.281880000	0.095926000	-0.664704000
O	-2.081012000	-0.873333000	0.218088000
O	-3.365082000	0.637336000	-0.797071000
O	0.351771000	-1.552919000	0.155553000
H	-1.089984000	-1.183024000	0.199478000
Au	1.078560000	6.223680000	1.001055000
Au	1.298057000	3.754594000	1.040379000

Glycine

N	1.957173000	-0.030662000	-0.000003000
C	0.720593000	0.717356000	0.000003000
C	-0.542249000	-0.117484000	0.000013000
O	-0.588870000	-1.324637000	0.000001000
H	1.981117000	-0.644023000	0.807867000
H	1.981108000	-0.644026000	-0.807872000

H	0.680972000	1.375073000	0.871768000
H	0.680962000	1.375071000	-0.871764000
O	-1.644628000	0.666275000	-0.000003000
H	-2.405667000	0.060553000	-0.000010000

Au^+-Glycine

N	-1.733138000	-1.695309000	0.096504000
C	-2.432377000	-0.621824000	-0.646235000
C	-2.151609000	0.747284000	-0.055813000
O	-1.231523000	1.002216000	0.693411000
H	-2.279364000	-1.957900000	0.917930000
H	-1.679406000	-2.529763000	-0.487188000
H	-2.071166000	-0.616029000	-1.676362000
H	-3.509320000	-0.795015000	-0.664830000
O	-3.037711000	1.620851000	-0.500970000
H	-2.812949000	2.504316000	-0.150971000
Au	0.170782000	-1.138211000	0.770806000

Au_2-Glycine

N	-0.807709000	0.991300000	0.116286000
C	-0.615355000	-0.378062000	0.618522000
C	-0.012627000	-1.196534000	-0.494708000
O	-0.175445000	-0.951826000	-1.666025000
H	-1.190185000	0.925534000	-0.828434000
H	-1.483228000	1.485614000	0.693975000
H	0.038438000	-0.377128000	1.487309000
H	-1.563689000	-0.855765000	0.891617000
O	0.672072000	-2.246291000	-0.021145000
H	0.992035000	-2.742633000	-0.795540000
Au	3.142498000	3.280172000	-0.032284000
Au	1.003198000	2.065617000	0.030425000

Histidine

C	0.834495000	10.545222000	-5.653854000
C	0.465073000	9.806794000	-6.925090000
N	-0.176418000	11.520125000	-5.261336000
O	-0.656436000	9.713940000	-7.368288000
O	1.539169000	9.218511000	-7.490803000
H	1.787362000	11.056847000	-5.811678000
H	-1.090682000	11.079419000	-5.351668000
H	-0.182369000	12.290481000	-5.924454000
H	1.207453000	8.729991000	-8.264782000
C	1.016528000	9.509835000	-4.532060000
C	1.622406000	10.097357000	-3.307816000
H	1.659861000	8.698305000	-4.878721000
H	0.030900000	9.075431000	-4.319679000
C	2.654177000	9.680880000	-2.489730000
N	2.842875000	10.557827000	-1.452768000
C	1.935459000	11.503543000	-1.637883000
N	1.174116000	11.261323000	-2.737706000
H	3.261497000	8.794732000	-2.599039000
H	1.794306000	12.372274000	-1.013446000

H	0.463319000	11.838899000	-3.173221000

Au^+-Histidine

N	-1.402242000	-1.776361000	1.968172000
C	-1.639491000	-0.946573000	0.906382000
C	-0.230483000	-2.414417000	1.779974000
N	0.290504000	-2.035262000	0.618934000
C	-0.571015000	-1.099087000	0.053225000
C	-0.361487000	-0.478863000	-1.300172000
Au	2.071409000	-1.753013000	-0.365335000
C	0.827784000	0.497854000	-1.510897000
N	2.152400000	-0.158069000	-1.744886000
C	1.003684000	1.443786000	-0.338654000
O	2.059691000	1.646838000	0.209282000
O	-0.157051000	2.029845000	-0.024868000
H	-2.015460000	-1.910724000	2.761834000
H	-2.524016000	-0.334503000	0.838575000
H	0.198007000	-3.124112000	2.468869000
H	-0.283933000	-1.269042000	-2.054821000
H	-1.267616000	0.084676000	-1.536820000
H	0.577609000	1.117905000	-2.379734000
H	2.884992000	0.520747000	-1.525533000
H	2.246741000	-0.429665000	-2.722101000
H	0.021973000	2.663035000	0.695571000

Au_2-Histidine

Au	3.041282000	13.333598000	-2.943441000
Au	3.641998000	15.470867000	-4.005377000
C	1.670890000	10.410226000	-5.230137000
C	0.567768000	9.404696000	-4.867605000
N	1.109078000	11.761722000	-5.238008000
O	0.786859000	8.225015000	-4.705069000
O	-0.631386000	9.962040000	-4.728628000
H	2.007694000	10.145388000	-6.236581000
H	1.354889000	12.261476000	-6.084987000
H	1.468225000	12.319542000	-4.461917000
H	-0.446459000	10.923871000	-4.918185000
C	2.843946000	10.198077000	-4.260655000
C	2.429477000	10.337369000	-2.837661000
H	3.636254000	10.916544000	-4.484460000
H	3.232368000	9.190949000	-4.420998000
C	1.895483000	9.383746000	-1.998099000
N	1.637167000	10.022150000	-0.816372000
C	1.995816000	11.317735000	-0.932639000
N	2.481201000	11.534456000	-2.152743000
H	1.671139000	8.343322000	-2.165691000
H	1.904494000	12.051658000	-0.149488000
H	1.239666000	9.598791000	0.009498000

Histidine(+H^+)

N	-1.057611000	-1.477122000	-0.313157000
C	-1.179597000	-0.116532000	0.235072000

C	-2.575674000	0.481236000	0.105265000
O	-2.807448000	1.606875000	-0.257694000
H	-1.670229000	-2.106923000	0.200909000
H	-1.393115000	-1.489287000	-1.275838000
H	-0.968028000	-0.182322000	1.308771000
C	-0.162266000	0.813039000	-0.424195000
C	1.235919000	0.368581000	-0.165954000
H	-0.300283000	1.830207000	-0.057428000
H	-0.351573000	0.845126000	-1.502830000
C	2.408848000	1.053002000	0.043334000
N	1.562769000	-0.965259000	-0.122168000
H	2.613912000	2.109577000	0.089428000
H	4.369015000	0.296909000	0.374954000
C	2.864216000	-1.120735000	0.104745000
N	3.389054000	0.106701000	0.203462000
H	3.399356000	-2.051060000	0.197763000
H	0.783910000	-1.653728000	-0.218985000
O	-3.507082000	-0.419212000	0.463262000
H	-4.376558000	0.015212000	0.386754000

Au_2-Histidine(+H^+)

N	-1.835833000	-1.743461000	-0.440727000
C	-1.201871000	-0.503507000	0.060510000
C	-2.184938000	0.629575000	0.342450000
O	-1.849897000	1.782442000	0.443274000
H	-2.554312000	-2.045301000	0.218690000
H	-2.326577000	-1.555301000	-1.316596000
H	-0.774425000	-0.752532000	1.036902000
C	-0.079567000	-0.012547000	-0.881753000
C	1.288486000	-0.077284000	-0.294569000
H	-0.265944000	1.033633000	-1.130497000
H	-0.108067000	-0.583762000	-1.812646000
C	2.040295000	0.939922000	0.245033000
N	2.062796000	-1.212169000	-0.194628000
H	1.816570000	1.987884000	0.361537000
H	3.994342000	0.884467000	1.083097000
C	3.228693000	-0.923602000	0.376562000
N	3.226544000	0.386395000	0.649454000
H	4.025437000	-1.620716000	0.579097000
H	1.788196000	-2.188058000	-0.483337000
O	-3.432543000	0.167803000	0.517216000
H	-3.999202000	0.928428000	0.746262000
Au	-0.326598000	-3.171150000	-0.704938000
Au	1.754187000	-4.461220000	-0.971459000

Isoleucine

N	0.593632000	1.685709000	-0.885586000
C	0.600100000	0.772512000	0.242938000
C	1.604361000	-0.353529000	0.082839000
O	2.186547000	-0.637300000	-0.938987000
H	1.446811000	2.236017000	-0.879730000
H	0.630608000	1.145199000	-1.745961000

H	0.885804000	1.328822000	1.141677000
C	-0.801418000	0.180818000	0.494131000
C	-1.763608000	1.294130000	0.889756000
H	-0.704190000	-0.518286000	1.332565000
C	-1.293316000	-0.599147000	-0.725500000
H	-0.515134000	-1.298185000	-1.051796000
H	-1.454347000	0.099777000	-1.552851000
C	-2.575543000	-1.381115000	-0.463571000
H	-2.451979000	-2.055498000	0.387227000
H	-2.847194000	-1.985515000	-1.330719000
H	-3.416137000	-0.720851000	-0.248577000
H	-1.381414000	1.849607000	1.749654000
H	-2.740352000	0.894193000	1.164065000
H	-1.889778000	1.996755000	0.065037000
O	1.770830000	-1.045041000	1.234526000
H	2.408044000	-1.751714000	1.030027000

Au^+-Isoleucine

C	-1.889587000	0.215281000	-0.633274000
C	-0.510749000	0.216814000	0.021175000
C	0.186635000	1.569742000	-0.202334000
C	0.361267000	-0.953949000	-0.438426000
C	1.541940000	1.628222000	0.476802000
N	0.362635000	1.864280000	-1.652086000
C	-0.227976000	-2.309771000	-0.063170000
O	2.584436000	1.889354000	-0.086114000
O	1.427967000	1.353432000	1.771684000
Au	1.622912000	3.431352000	-2.089168000
H	-1.824572000	0.122021000	-1.721816000
H	-2.450768000	1.122029000	-0.391957000
H	-2.480694000	-0.629181000	-0.282425000
H	-0.638875000	0.140826000	1.105569000
H	-0.430342000	2.371467000	0.215076000
H	1.359157000	-0.867977000	0.004610000
H	0.496352000	-0.924962000	-1.528001000
H	-0.558836000	2.005186000	-2.068203000
H	0.760735000	1.037605000	-2.104829000
H	-0.419560000	-2.362660000	1.010267000
H	0.464485000	-3.111492000	-0.319140000
H	-1.163547000	-2.504200000	-0.586874000
H	2.316988000	1.404579000	2.171634000

Au_2-Isoleucine

N	0.584968000	1.715526000	-0.862518000
C	0.545303000	0.819015000	0.307795000
C	1.598848000	-0.257300000	0.130844000
O	2.157100000	-0.508224000	-0.913279000
H	1.524539000	2.108404000	-0.929459000
H	0.487404000	1.142886000	-1.702202000
H	0.801519000	1.406283000	1.193343000
C	-0.832119000	0.158210000	0.526476000
C	-1.823483000	1.079872000	1.230901000

H	-0.634939000	-0.684999000	1.198405000
C	-1.385383000	-0.399190000	-0.785631000
H	-0.603328000	-0.966556000	-1.305400000
H	-1.666597000	0.436384000	-1.435987000
C	-2.596063000	-1.302683000	-0.582844000
H	-2.361083000	-2.122395000	0.100240000
H	-2.916087000	-1.738111000	-1.530444000
H	-3.442176000	-0.751668000	-0.172214000
H	-1.363189000	1.567388000	2.093364000
H	-2.679070000	0.507816000	1.592745000
H	-2.194480000	1.861789000	0.564412000
O	1.833052000	-0.916198000	1.279781000
H	2.499762000	-1.596562000	1.077291000
Au	-0.769686000	3.308546000	-0.917347000
Au	-2.370971000	5.176522000	-1.012214000

Leucine

N	-0.574136000	-1.801831000	-0.029790000
C	-0.609884000	-0.394699000	0.317850000
C	-1.927515000	0.275787000	-0.016277000
O	-2.738882000	-0.134347000	-0.814124000
H	-1.299189000	-2.300035000	0.478124000
H	-0.813697000	-1.900537000	-1.013779000
H	-0.452376000	-0.290346000	1.394396000
C	0.494530000	0.366017000	-0.423992000
C	2.259585000	-0.223141000	1.281932000
H	0.264589000	0.340508000	-1.497149000
C	1.899310000	-0.188293000	-0.197522000
H	1.925429000	-1.212295000	-0.580383000
H	0.464206000	1.416766000	-0.115852000
C	2.903296000	0.655579000	-0.975146000
H	2.653058000	0.695719000	-2.037977000
H	3.912960000	0.249821000	-0.882538000
H	2.919647000	1.681627000	-0.595894000
H	1.635390000	-0.928600000	1.832465000
H	2.141800000	0.767797000	1.732232000
H	3.299291000	-0.528861000	1.418183000
O	-2.087872000	1.433740000	0.665889000
H	-2.929166000	1.811753000	0.355264000

Au^+-Leucine

C	-1.282738000	-0.753583000	-2.233625000
C	0.134779000	-0.894482000	-1.688887000
C	0.152815000	-1.009815000	-0.163224000
C	0.816777000	-2.123304000	-2.282086000
C	-0.563550000	0.125375000	0.569380000
C	-0.433952000	-0.024599000	2.071482000
N	-0.002708000	1.448731000	0.176621000
O	0.083509000	0.799730000	2.795482000
O	-0.948220000	-1.180589000	2.475304000
Au	-0.603179000	3.036890000	1.329725000
H	-1.274504000	-0.754283000	-3.324325000

H	-1.781710000	0.169388000	-1.921824000
H	-1.903434000	-1.591764000	-1.905295000
H	0.724079000	-0.018448000	-1.993054000
H	1.187811000	-1.065538000	0.197382000
H	-0.334454000	-1.942931000	0.133551000
H	1.830117000	-2.245554000	-1.894986000
H	0.877774000	-2.046340000	-3.368180000
H	0.250155000	-3.025758000	-2.040165000
H	-1.625889000	0.133174000	0.314802000
H	-0.209909000	1.606735000	-0.810928000
H	1.017431000	1.405246000	0.253682000
H	-0.830999000	-1.244284000	3.442166000

Au_2-Leucine

N	-0.516673000	-1.894689000	-0.198772000
C	-0.500738000	-0.506726000	0.301258000
C	-1.871328000	0.109914000	0.111224000
O	-2.776734000	-0.405933000	-0.503887000
H	-1.423752000	-2.306719000	0.025375000
H	-0.490545000	-1.867862000	-1.219032000
H	-0.290654000	-0.526669000	1.373084000
C	0.547906000	0.337614000	-0.422158000
C	2.387191000	-0.001499000	1.271150000
H	0.314280000	0.319366000	-1.495627000
C	2.005178000	-0.067008000	-0.201393000
H	2.151418000	-1.096108000	-0.547872000
H	0.421410000	1.373240000	-0.091057000
C	2.898974000	0.849110000	-1.029990000
H	2.644318000	0.805266000	-2.091663000
H	3.947468000	0.565244000	-0.923883000
H	2.797376000	1.887662000	-0.700901000
H	1.834905000	-0.723724000	1.875852000
H	2.206992000	1.002283000	1.670278000
H	3.446410000	-0.232891000	1.399271000
O	-1.962685000	1.307361000	0.714829000
H	-2.864640000	1.633525000	0.546706000
Au	0.993765000	-3.147587000	0.542998000
Au	2.781331000	-4.582608000	1.438259000

Lysine

N	-1.172716000	-1.787683000	0.106709000
C	-1.331892000	-0.378187000	0.414627000
C	-2.662868000	0.184504000	-0.042460000
O	-3.355830000	-0.286751000	-0.914824000
H	-1.834150000	-2.335541000	0.648642000
H	-1.429123000	-1.933906000	-0.867190000
H	-1.260551000	-0.239297000	1.497484000
C	-0.220527000	0.437125000	-0.255999000
H	-0.419657000	1.503206000	-0.113349000
H	-0.261831000	0.243156000	-1.335110000
C	1.160064000	0.087812000	0.279869000
H	1.304662000	-0.990171000	0.187320000

H	1.198104000	0.325053000	1.350482000
C	2.269213000	0.840906000	-0.441186000
H	2.256065000	0.580135000	-1.506590000
C	3.659823000	0.555159000	0.124693000
H	2.079384000	1.919253000	-0.381155000
H	3.686378000	0.845948000	1.179455000
H	4.393734000	1.183738000	-0.386859000
N	4.122854000	-0.826774000	0.036356000
H	3.508659000	-1.438161000	0.563035000
H	4.087815000	-1.142024000	-0.928290000
H	-3.815513000	1.634778000	0.224105000
O	-2.981110000	1.322629000	0.615980000

Au^+-Lysine

N	-1.016722000	1.686508000	0.961972000
C	-0.680130000	1.786327000	-0.494991000
Au	-0.350792000	-0.073940000	1.707964000
C	-0.630399000	0.431684000	-1.218516000
C	0.639365000	2.530111000	-0.580384000
N	0.412117000	-1.931493000	1.949544000
C	0.652808000	-0.405345000	-0.990036000
O	1.205351000	2.997184000	0.380564000
C	1.159407000	-2.317145000	0.699408000
O	1.066510000	2.602798000	-1.841472000
C	0.436631000	-1.883284000	-0.576266000
H	-0.557296000	2.474133000	1.432583000
H	-2.022941000	1.792407000	1.088743000
H	-1.432219000	2.417720000	-0.979924000
H	-1.523325000	-0.129455000	-0.927517000
H	-0.743183000	0.643663000	-2.283639000
H	-0.345710000	-2.598876000	2.100797000
H	1.027717000	-2.022801000	2.758539000
H	1.234785000	-0.386363000	-1.913615000
H	1.295734000	0.084187000	-0.248970000
H	1.303643000	-3.399471000	0.711847000
H	2.141177000	-1.845152000	0.760704000
H	1.891737000	3.123281000	-1.846336000
H	-0.629925000	-2.108824000	-0.472873000
H	0.794790000	-2.536372000	-1.375500000

Au_2-Lysine

N	-1.267151000	2.021158000	0.718321000
C	-0.792385000	1.890506000	-0.642535000
Au	0.758702000	-0.947172000	2.824694000
C	-0.704237000	0.412454000	-1.041307000
C	0.545169000	2.561504000	-0.902999000
N	1.036293000	-2.726731000	1.746626000
C	0.447376000	-0.307927000	-0.353172000
O	1.264680000	3.062179000	-0.072872000
C	1.401490000	-2.589437000	0.309210000
O	0.863719000	2.527865000	-2.224179000
C	0.343806000	-1.822717000	-0.474794000

H	-0.592050000	1.644194000	1.383236000
H	-1.353705000	3.004094000	0.959510000
H	-1.516013000	2.374051000	-1.307607000
H	-1.656006000	-0.041047000	-0.750737000
H	-0.605891000	0.329357000	-2.127026000
H	0.185872000	-3.285489000	1.811222000
H	1.761015000	-3.263996000	2.221363000
H	1.403482000	0.020793000	-0.776506000
H	0.471998000	-0.034090000	0.706422000
H	1.536913000	-3.588000000	-0.118178000
H	2.360399000	-2.069711000	0.268254000
H	1.723376000	2.978030000	-2.296922000
H	-0.649357000	-2.153510000	-0.147214000
H	0.428845000	-2.108436000	-1.528132000
Au	0.449319000	1.207768000	3.975214000

Lysine(+H$^+$)

N	0.238418000	0.119950000	1.767186000
C	1.116647000	-0.599169000	0.830011000
C	1.709536000	0.382397000	-0.157343000
O	1.233850000	1.466393000	-0.436362000
H	0.796589000	0.772393000	2.317392000
H	-0.136267000	-0.552066000	2.435072000
H	1.951765000	-1.083474000	1.346238000
C	0.359855000	-1.684493000	0.050023000
H	-0.052748000	-2.397796000	0.771479000
H	1.110020000	-2.233305000	-0.523950000
C	-0.748773000	-1.214319000	-0.897809000
H	-0.930281000	-2.030199000	-1.599348000
H	-0.385921000	-0.388186000	-1.520219000
C	-2.088914000	-0.846294000	-0.227047000
H	-2.891637000	-1.458627000	-0.642899000
C	-2.527145000	0.597214000	-0.401549000
H	-2.061789000	-1.081953000	0.839812000
H	-3.464822000	0.777417000	0.123210000
H	-2.668355000	0.853809000	-1.451522000
N	-1.508483000	1.536807000	0.168518000
H	-0.949530000	1.054780000	0.947249000
H	-0.769608000	1.778107000	-0.500536000
H	3.128205000	0.531930000	-1.378373000
O	2.804830000	-0.115612000	-0.724237000
H	-1.929173000	2.404762000	0.499241000

Au_2-Lysine(+H$^+$)

N	0.783532000	-0.519651000	2.267407000
C	1.270594000	-0.805557000	0.889154000
C	1.574647000	0.485640000	0.152902000
O	0.811601000	1.423712000	0.035177000
H	1.578035000	-0.191824000	2.820864000
H	0.522005000	-1.415593000	2.683385000
H	2.212231000	-1.352574000	0.979011000
C	0.315778000	-1.690342000	0.056538000

H	-0.109987000	-2.446741000	0.726022000
H	0.963610000	-2.237601000	-0.632992000
C	-0.798640000	-1.038678000	-0.771644000
H	-1.045319000	-1.737288000	-1.575130000
H	-0.401475000	-0.157893000	-1.283056000
C	-2.091984000	-0.702485000	-0.017431000
H	-2.779554000	-1.550366000	-0.064858000
C	-2.812853000	0.511237000	-0.576337000
H	-1.900987000	-0.526270000	1.044533000
H	-3.816372000	0.604958000	-0.160188000
H	-2.885591000	0.471968000	-1.664417000
N	-2.086062000	1.767623000	-0.206133000
H	-2.245773000	1.973765000	0.825512000
H	-1.064158000	1.680067000	-0.314245000
H	2.916671000	1.263470000	-0.908573000
O	2.781130000	0.437830000	-0.405182000
H	-2.414415000	2.572878000	-0.742282000
Au	-0.785919000	0.818351000	2.615648000
Au	-2.695331000	2.349861000	2.927848000

Methionine

N	-1.697223000	1.416741000	0.673927000
C	-1.318745000	0.617244000	-0.479030000
C	-1.115498000	-0.872754000	-0.232668000
O	-0.607412000	-1.637992000	-1.022784000
H	-2.606001000	1.116104000	1.011348000
H	-2.156579000	0.644791000	-1.186755000
C	-0.111603000	1.220343000	-1.198380000
C	1.018969000	1.623805000	-0.260239000
H	-0.444607000	2.120956000	-1.722374000
H	0.239997000	0.511751000	-1.950809000
H	0.682120000	2.422454000	0.404557000
H	1.864248000	2.015376000	-0.833210000
S	1.617685000	0.316462000	0.835446000
C	2.426632000	-0.755092000	-0.367677000
H	2.930032000	-1.540294000	0.196670000
H	3.174190000	-0.192370000	-0.928883000
H	1.703773000	-1.212002000	-1.042300000
H	-1.050456000	1.229785000	1.435727000
O	-1.672362000	-1.289261000	0.925931000
H	-1.522481000	-2.250344000	0.951289000

Au^+-Methionine

N	-0.670883000	2.142370000	0.602532000
C	-1.056533000	1.197757000	-0.499070000
Au	0.827399000	1.048536000	1.568727000
C	-2.168122000	0.324056000	0.056949000
C	0.089088000	0.368288000	-1.134982000
S	1.786425000	-0.883465000	0.916959000
O	-2.640294000	0.464519000	1.159057000
O	-2.553838000	-0.585201000	-0.842584000
C	0.565411000	-0.940194000	-0.471731000

C	3.300886000	-0.456972000	0.028381000
H	-1.501608000	2.299675000	1.180285000
H	-0.373090000	3.036997000	0.216596000
H	-1.506435000	1.779330000	-1.312235000
H	0.933978000	1.032779000	-1.339418000
H	-0.294051000	0.067447000	-2.115723000
H	-3.306295000	-1.078779000	-0.464187000
H	1.030978000	-1.574262000	-1.232057000
H	-0.272639000	-1.506921000	-0.059482000
H	3.174122000	0.432903000	-0.583867000
H	3.579385000	-1.313799000	-0.585103000
H	4.069151000	-0.281261000	0.780353000

Au_2-Methionine

N	-1.473726000	1.593490000	0.870429000
C	-1.224206000	0.584449000	-0.148684000
C	-1.062545000	-0.844848000	0.358786000
O	-0.348459000	-1.692621000	-0.135352000
H	-2.392134000	1.440676000	1.276194000
H	-2.127902000	0.526393000	-0.768830000
C	-0.089957000	1.001671000	-1.087322000
C	1.093680000	1.716903000	-0.452965000
H	-0.508495000	1.725462000	-1.793234000
H	0.236445000	0.138736000	-1.670412000
H	0.748085000	2.537228000	0.180228000
H	1.720648000	2.146935000	-1.239726000
S	2.240247000	0.781178000	0.596058000
C	2.668129000	-0.599519000	-0.480649000
H	3.436430000	-1.168072000	0.042364000
H	3.079853000	-0.199648000	-1.408768000
H	1.801439000	-1.230035000	-0.667255000
H	-0.819030000	1.456547000	1.640699000
O	-1.908400000	-1.087421000	1.372558000
H	-1.701673000	-1.978106000	1.709403000
Au	1.117664000	-0.073967000	2.360401000
Au	-0.055711000	-0.983552000	4.336701000

Phenylalanine

N	-1.395835000	-1.862320000	-0.554494000
C	-1.700111000	-0.796764000	0.376667000
C	-1.992764000	0.531556000	-0.299931000
O	-1.862742000	0.766330000	-1.477495000
H	-0.607789000	-1.576963000	-1.131934000
H	-2.173720000	-1.984953000	-1.196341000
H	-2.596619000	-1.067305000	0.944276000
C	-0.547583000	-0.627342000	1.385233000
C	0.736195000	-0.245517000	0.705827000
H	-0.433584000	-1.584724000	1.898467000
H	-0.828718000	0.127278000	2.122718000
C	3.086204000	0.466642000	-0.653756000
C	1.620456000	-1.227598000	0.247543000
C	1.051953000	1.098843000	0.481348000

C	2.216552000	1.453938000	-0.194254000
C	2.786237000	-0.875383000	-0.429642000
H	1.388415000	-2.272969000	0.425491000
H	0.381826000	1.870992000	0.847352000
H	2.448223000	2.500636000	-0.357441000
H	3.463137000	-1.648643000	-0.775865000
H	3.994256000	0.741861000	-1.178073000
O	-2.434480000	1.456261000	0.589126000
H	-2.599012000	2.263067000	0.070085000

Au^+-Phenylalanine

N	-0.039939000	2.210672000	1.105622000
C	0.725267000	1.969264000	-0.150157000
Au	-1.470282000	0.586498000	1.232387000
C	1.473438000	0.607753000	-0.216608000
C	-0.252710000	2.190089000	-1.293545000
C	-0.636706000	-0.803785000	-0.201850000
C	0.680226000	-0.565908000	0.289994000
O	-1.374667000	2.613138000	-1.139433000
C	-1.538271000	-1.621701000	0.547413000
O	0.302844000	1.900032000	-2.473245000
C	1.124906000	-1.320908000	1.369474000
C	-1.052949000	-2.338448000	1.668687000
C	0.279558000	-2.225076000	2.032280000
H	-0.513324000	3.111892000	1.024086000
H	0.596390000	2.262769000	1.900908000
H	1.489910000	2.747415000	-0.266977000
H	1.779962000	0.479126000	-1.257878000
H	2.388934000	0.690868000	0.374300000
H	-0.940766000	-0.421437000	-1.173573000
H	-2.512355000	-1.855943000	0.130006000
H	-0.337964000	2.140262000	-3.169115000
H	2.136291000	-1.172155000	1.735533000
H	-1.722753000	-2.994433000	2.212298000
H	0.663962000	-2.808984000	2.860390000

Au_2-Phenylalanine

N	-1.366360000	-1.787682000	-0.540597000
C	-1.721490000	-0.780417000	0.468848000
C	-2.093831000	0.520891000	-0.213709000
O	-2.046150000	0.710966000	-1.406156000
H	-0.545457000	-1.448769000	-1.048900000
H	-2.112801000	-1.841610000	-1.233770000
H	-2.600077000	-1.135360000	1.014992000
C	-0.565575000	-0.588328000	1.464375000
C	0.721819000	-0.273684000	0.756128000
H	-0.465073000	-1.511792000	2.039494000
H	-0.841133000	0.212712000	2.153675000
C	3.073942000	0.286134000	-0.662938000
C	1.639411000	-1.291442000	0.472279000
C	1.002550000	1.029132000	0.328767000
C	2.170145000	1.308257000	-0.376781000

C	2.809973000	-1.011767000	-0.230696000
H	1.441644000	-2.301277000	0.820784000
H	0.310499000	1.831991000	0.565598000
H	2.376984000	2.322892000	-0.698235000
H	3.513820000	-1.809747000	-0.439186000
H	3.984175000	0.503136000	-1.210228000
O	-2.506442000	1.444260000	0.676551000
H	-2.747662000	2.234015000	0.160062000
Au	-0.948481000	-3.672638000	0.273996000
Au	-0.436812000	-5.860453000	1.279480000

Proline

N	-0.816684000	1.001758000	0.700558000
C	0.063691000	-0.161607000	0.687264000
C	1.393392000	0.118841000	0.029209000
O	1.697335000	1.140865000	-0.542797000
H	-0.267843000	1.850197000	0.591252000
C	-1.765047000	0.837263000	-0.404707000
C	-0.702982000	-1.256042000	-0.102746000
H	-0.223534000	-1.425954000	-1.069692000
C	-2.089816000	-0.643822000	-0.309961000
H	-0.715090000	-2.206453000	0.429145000
H	-2.722728000	-0.821352000	0.561679000
H	-2.592430000	-1.034217000	-1.195576000
H	-1.322674000	1.063180000	-1.386237000
H	-2.632445000	1.482722000	-0.257896000
H	0.275001000	-0.497993000	1.707127000
O	2.230594000	-0.936245000	0.147421000
H	3.051455000	-0.680628000	-0.308216000

Au^+-Proline

N	-0.742976000	0.995714000	0.819317000
C	0.105684000	-0.239290000	0.733089000
C	1.474980000	0.034831000	0.145019000
O	1.877727000	1.103456000	-0.264473000
H	-1.252253000	0.934951000	1.703051000
C	-1.770655000	0.868178000	-0.272461000
C	-0.684520000	-1.232515000	-0.158681000
H	-0.224398000	-1.291714000	-1.146826000
C	-2.078556000	-0.616403000	-0.266378000
H	-0.677543000	-2.232132000	0.271575000
H	-2.694035000	-0.871750000	0.600105000
H	-2.606573000	-0.935304000	-1.164684000
H	-1.306002000	1.172440000	-1.211016000
H	-2.608643000	1.527886000	-0.051283000
H	0.248444000	-0.629059000	1.743238000
O	2.171763000	-1.094801000	0.115548000
H	3.044192000	-0.909759000	-0.281257000
Au	0.275152000	2.795121000	0.832506000

Au_2-Proline

N	-0.815455000	1.029925000	0.854979000

C	0.092022000	-0.132592000	0.802541000
C	1.268378000	0.167851000	-0.105457000
O	1.500222000	1.248376000	-0.598376000
H	-0.265591000	1.890789000	0.820141000
C	-1.635969000	0.896060000	-0.378242000
C	-0.804639000	-1.279958000	0.301438000
H	-0.258057000	-1.923922000	-0.386338000
C	-2.011206000	-0.579868000	-0.363525000
H	-1.127001000	-1.885544000	1.147517000
H	-2.908564000	-0.728520000	0.237313000
H	-2.204396000	-0.952620000	-1.369479000
H	-1.023866000	1.144413000	-1.251349000
H	-2.489390000	1.569986000	-0.330991000
H	0.501542000	-0.340421000	1.793615000
O	2.056664000	-0.909797000	-0.260388000
H	2.808849000	-0.625713000	-0.809871000
Au	-1.989905000	0.941212000	2.587575000
Au	-3.374596000	0.789958000	4.617556000

Serine

N	0.784449000	1.738390000	-0.046467000
C	-0.057280000	0.601120000	0.323989000
C	0.724268000	-0.691721000	0.085211000
O	0.195210000	-1.777781000	-0.015292000
H	0.664501000	1.967670000	-1.029708000
H	0.545534000	2.563703000	0.490358000
H	-0.244821000	0.644357000	1.401700000
C	-1.407751000	0.547730000	-0.389325000
O	-2.252342000	-0.450083000	0.128373000
H	-1.231375000	0.414465000	-1.467588000
H	-1.916034000	1.504235000	-0.249236000
H	-1.738435000	-1.273993000	0.076691000
O	2.044037000	-0.524355000	0.010768000
H	2.162823000	0.454574000	0.064988000

Au^+-Serine

N	0.200391000	0.434398000	-1.760311000
C	0.618114000	0.236031000	-0.349266000
C	2.118542000	-0.030931000	-0.284356000
O	2.889926000	0.110137000	-1.208147000
H	0.072797000	-0.503572000	-2.158182000
H	-0.714656000	0.885665000	-1.776564000
H	0.419781000	1.153537000	0.210976000
C	-0.169804000	-0.928886000	0.233975000
O	0.098119000	-2.000720000	-0.654555000
H	-1.236421000	-0.678834000	0.257459000
H	0.170792000	-1.131199000	1.250500000
H	-0.305498000	-2.813675000	-0.322024000
O	2.467385000	-0.407701000	0.938481000
H	3.433349000	-0.547872000	0.949709000
Au	1.541956000	1.454886000	-2.967748000

Au$_2$-Serine

N	0.080872000	0.260821000	-1.650186000
C	0.484743000	0.049668000	-0.247127000
C	1.922693000	-0.414092000	-0.263602000
O	2.287097000	-1.400922000	-0.868409000
H	0.084670000	-0.650465000	-2.112696000
H	-0.884609000	0.588623000	-1.670288000
H	0.411489000	0.996366000	0.287823000
C	-0.370501000	-1.026233000	0.431999000
O	-0.449993000	-2.201537000	-0.345347000
H	-1.392964000	-0.656888000	0.535808000
H	0.022204000	-1.220378000	1.437580000
H	0.455433000	-2.522369000	-0.482375000
O	2.713744000	0.330962000	0.512948000
H	3.611895000	-0.041714000	0.441448000
Au	2.905261000	3.202262000	-3.687766000
Au	1.360377000	1.614157000	-2.617676000

Threonine

N	-0.062123000	1.884655000	0.260471000
C	0.005166000	0.440789000	0.450273000
C	1.310082000	-0.147814000	-0.031155000
O	1.903538000	0.231429000	-1.016101000
H	0.481297000	2.365846000	0.969119000
H	0.354638000	2.114742000	-0.639308000
H	-0.142317000	0.206399000	1.506565000
C	-1.164901000	-0.175119000	-0.336432000
O	-2.361273000	0.426582000	0.113600000
C	-1.297702000	-1.669507000	-0.158550000
H	-0.995562000	0.060992000	-1.399192000
H	-2.139632000	1.370493000	0.200204000
H	-0.442731000	-2.200186000	-0.578655000
H	-2.200104000	-2.011240000	-0.665630000
H	-1.381088000	-1.918795000	0.900581000
O	1.737489000	-1.168199000	0.741490000
H	2.553771000	-1.498413000	0.325009000

Au$^+$-Threonine

N	0.362665000	1.504491000	0.246843000
C	0.567674000	0.067702000	0.558706000
C	1.867835000	-0.344872000	-0.110838000
O	2.550872000	0.419826000	-0.749901000
H	-0.330716000	1.908813000	0.875423000
H	1.247857000	1.994359000	0.397373000
H	0.680888000	-0.058548000	1.640782000
C	-0.593760000	-0.806250000	0.089093000
O	-0.565848000	-0.749198000	-1.339146000
C	-1.930351000	-0.369895000	0.654086000
H	-0.356811000	-1.821898000	0.418429000
H	-1.190337000	-1.398644000	-1.694349000
H	-1.913112000	-0.378940000	1.746166000
H	-2.715840000	-1.055659000	0.333910000

H	-2.206485000	0.627936000	0.306588000
O	2.137160000	-1.625111000	0.126711000
H	2.982986000	-1.842113000	-0.308784000
Au	-0.115840000	1.803956000	-1.751523000

Au_2-Threonine

N	0.454185000	1.387139000	0.453056000
C	0.609025000	-0.065894000	0.679173000
C	1.909186000	-0.484273000	0.028422000
O	2.692206000	0.285882000	-0.475845000
H	-0.064949000	1.797081000	1.226341000
H	1.392351000	1.793846000	0.452329000
H	0.712407000	-0.265846000	1.753923000
C	-0.560726000	-0.918596000	0.159014000
O	-0.532651000	-1.063133000	-1.243820000
C	-1.898335000	-0.414509000	0.672713000
H	-0.379365000	-1.923799000	0.546305000
H	-0.750107000	-0.195047000	-1.640657000
H	-1.907141000	-0.361649000	1.765135000
H	-2.686721000	-1.095729000	0.353348000
H	-2.124157000	0.573889000	0.266731000
O	2.104914000	-1.805341000	0.132057000
H	2.946627000	-1.995946000	-0.318332000
Au	-1.491135000	2.720490000	-3.457720000
Au	-0.425611000	2.031432000	-1.352173000

Tryptophan

N	-1.386666000	-2.051163000	-0.347705000
C	-2.128630000	-1.042640000	0.378825000
C	-2.715591000	0.036986000	-0.514446000
O	-2.554811000	0.132800000	-1.707642000
H	-1.976676000	-2.451117000	-1.071961000
H	-0.616423000	-1.602567000	-0.839905000
H	-2.971343000	-1.523436000	0.888610000
C	-1.236437000	-0.388429000	1.447858000
C	-0.112144000	0.386896000	0.845030000
H	-0.850139000	-1.191273000	2.081187000
H	-1.850956000	0.264479000	2.071125000
C	-0.117457000	1.736996000	0.556834000
C	1.156665000	-0.110422000	0.406826000
H	-0.887181000	2.472922000	0.740674000
N	1.071261000	2.092785000	-0.031374000
C	1.877269000	0.987971000	-0.136326000
H	1.315717000	3.023123000	-0.330099000
C	1.768000000	-1.376753000	0.438101000
C	3.169043000	0.856124000	-0.650258000
H	3.704702000	1.703918000	-1.064824000
C	3.744071000	-0.404905000	-0.608714000
H	4.745676000	-0.545672000	-0.999176000
C	3.050276000	-1.507282000	-0.069898000
H	1.237253000	-2.234318000	0.837611000
H	3.534731000	-2.477239000	-0.053952000

O	-3.476056000	0.910992000	0.193606000
H	-3.820043000	1.545605000	-0.459076000

Au^+-Tryptophan

Au	0.842101000	-1.495309000	1.178168000
N	2.500299000	-0.277653000	1.501764000
C	-0.352132000	-2.197600000	-0.444662000
C	2.748863000	0.559987000	0.279038000
C	-0.410462000	-0.773791000	-0.697514000
C	-1.521385000	-2.834444000	0.026831000
C	2.347118000	1.977073000	0.648406000
C	2.054469000	0.104081000	-1.035350000
C	0.556715000	0.232779000	-1.039984000
C	-1.673407000	-0.122182000	-0.565408000
C	-2.722954000	-2.134390000	0.189319000
O	1.879748000	2.282715000	1.720144000
O	2.603259000	2.829730000	-0.348053000
C	-0.158797000	1.410847000	-1.164020000
N	-1.481531000	1.190308000	-0.886022000
C	-2.825586000	-0.789915000	-0.151284000
H	2.335847000	0.378243000	2.274640000
H	3.334546000	-0.817092000	1.726418000
H	0.426533000	-2.810860000	-0.900327000
H	3.826732000	0.574857000	0.085849000
H	-1.495584000	-3.905396000	0.196340000
H	2.360194000	-0.924382000	-1.246096000
H	2.487336000	0.720558000	-1.828223000
H	-3.599798000	-2.661815000	0.544629000
H	2.392016000	3.726429000	-0.026475000
H	0.190377000	2.398962000	-1.424303000
H	-2.201855000	1.898541000	-0.903462000
H	-3.775661000	-0.271277000	-0.073362000

Au_2-Tryptophan

N	-1.420412000	-1.934275000	-0.457304000
C	-2.165377000	-1.033928000	0.434273000
C	-2.853538000	0.040712000	-0.383096000
O	-2.810496000	0.125558000	-1.587920000
H	-2.012838000	-2.184084000	-1.249499000
H	-0.638361000	-1.404610000	-0.854081000
H	-2.939433000	-1.614354000	0.945142000
C	-1.221192000	-0.415389000	1.475984000
C	-0.108419000	0.334314000	0.822001000
H	-0.826544000	-1.225524000	2.095233000
H	-1.810633000	0.238274000	2.122148000
C	-0.154057000	1.641244000	0.373387000
C	1.185131000	-0.169714000	0.467565000
H	-0.946553000	2.370825000	0.460613000
N	1.030004000	1.960330000	-0.239392000
C	1.873929000	0.878401000	-0.201448000
H	1.252356000	2.858701000	-0.638073000
C	1.844137000	-1.396476000	0.673583000

C	3.177197000	0.732607000	-0.682026000
H	3.688058000	1.541735000	-1.193501000
C	3.794549000	-0.491911000	-0.477616000
H	4.805991000	-0.643277000	-0.837516000
C	3.137225000	-1.539368000	0.198590000
H	1.363163000	-2.211824000	1.205251000
H	3.653701000	-2.480921000	0.347930000
O	-3.550613000	0.888596000	0.399852000
H	-3.992171000	1.513071000	-0.203038000
Au	-0.685239000	-3.630581000	0.522210000
Au	0.198889000	-5.577838000	1.741929000

Tyrosine

N	-1.843868000	-1.764970000	-0.736879000
C	-2.158920000	-0.741917000	0.237844000
C	-2.293329000	0.645082000	-0.366557000
O	-2.044002000	0.942399000	-1.510359000
H	-0.987273000	-1.500959000	-1.219102000
H	-2.564152000	-1.784328000	-1.453152000
H	-3.121191000	-0.979780000	0.703333000
C	-1.094878000	-0.725722000	1.351824000
C	0.269320000	-0.394911000	0.819105000
H	-1.094211000	-1.720897000	1.802136000
H	-1.391397000	-0.004372000	2.116112000
C	2.781980000	0.236018000	-0.268627000
C	0.703338000	0.932606000	0.726044000
C	1.125892000	-1.400436000	0.364086000
C	2.371633000	-1.092635000	-0.178189000
C	1.944112000	1.251608000	0.188050000
H	0.061569000	1.729879000	1.088701000
H	0.814694000	-2.437872000	0.434785000
H	3.026667000	-1.888511000	-0.521953000
H	2.281086000	2.279571000	0.120299000
O	3.991993000	0.605310000	-0.785153000
H	4.462762000	-0.191888000	-1.062438000
O	-2.742000000	1.544286000	0.544811000
H	-2.800149000	2.392518000	0.070934000

Au^+-Tyrosine

N	2.485668000	0.510660000	-1.106892000
C	2.484994000	0.676980000	0.375927000
Au	0.872854000	-0.864244000	-1.544298000
C	1.164274000	1.231161000	0.982945000
C	2.912765000	-0.664571000	0.950860000
C	-0.226334000	-0.782154000	0.322357000
C	-0.085890000	0.629546000	0.402022000
O	3.311539000	-1.583371000	0.273978000
C	-1.143252000	-1.367300000	-0.607969000
O	2.834163000	-0.672646000	2.284579000
C	-1.012153000	1.411022000	-0.282150000
C	-2.060724000	-0.523215000	-1.292143000
C	-2.011944000	0.852008000	-1.087296000

O	-2.949822000	-1.149409000	-2.081456000
H	3.388845000	0.122817000	-1.384117000
H	2.383029000	1.417937000	-1.561240000
H	3.277309000	1.380235000	0.661879000
H	1.239510000	1.076451000	2.062207000
H	1.139600000	2.311501000	0.819890000
H	0.317364000	-1.433004000	1.003077000
H	-1.334401000	-2.435906000	-0.596746000
H	3.201504000	-1.521219000	2.596840000
H	-0.938313000	2.492660000	-0.219770000
H	-2.736030000	1.497217000	-1.574837000
H	-3.539556000	-0.506161000	-2.503651000

Au_2-Tyrosine

N	-1.769929000	-1.612471000	-0.928718000
C	-2.175449000	-0.774324000	0.208689000
C	-2.419738000	0.644716000	-0.264599000
O	-2.254245000	1.030619000	-1.397834000
H	-0.893112000	-1.234615000	-1.297138000
H	-2.451047000	-1.505727000	-1.680443000
H	-3.118464000	-1.162166000	0.604326000
C	-1.112165000	-0.829138000	1.318274000
C	0.255255000	-0.502622000	0.790064000
H	-1.122510000	-1.837934000	1.737640000
H	-1.408207000	-0.133090000	2.106201000
C	2.773373000	0.095797000	-0.296795000
C	0.653134000	0.823789000	0.582887000
C	1.147295000	-1.523918000	0.449960000
C	2.398783000	-1.230155000	-0.085562000
C	1.898032000	1.126321000	0.045006000
H	-0.012014000	1.635358000	0.863016000
H	0.869872000	-2.559862000	0.622813000
H	3.080856000	-2.037252000	-0.337998000
H	2.209399000	2.152720000	-0.110729000
O	3.981343000	0.450729000	-0.820920000
H	4.487580000	-0.352617000	-1.002749000
O	-2.865332000	1.428772000	0.736355000
H	-3.018493000	2.308600000	0.347630000
Au	-1.483048000	-3.626536000	-0.431386000
Au	-1.093772000	-5.970822000	0.211839000

Tyrosine(-H^+)

N	-1.392154000	-1.086407000	1.319246000
C	-1.715548000	0.056841000	0.484608000
C	-3.163265000	-0.000268000	0.105831000
O	-3.814777000	-1.007558000	-0.071622000
H	-0.373981000	-1.160960000	1.332503000
H	-1.736287000	-1.923208000	0.853151000
H	-1.532961000	0.976224000	1.046475000
C	-0.892549000	0.141916000	-0.835665000
C	0.575861000	0.118403000	-0.556326000
H	-1.187049000	1.055128000	-1.367084000

H	-1.175782000	-0.716029000	-1.458884000
C	3.400829000	0.038088000	0.174722000
C	1.251023000	1.263440000	-0.103262000
C	1.324207000	-1.066142000	-0.638658000
C	2.669551000	-1.111899000	-0.295181000
C	2.593846000	1.233331000	0.242368000
H	0.703806000	2.204629000	-0.030565000
H	0.834588000	-1.972485000	-0.997456000
H	3.223144000	-2.043828000	-0.384395000
H	3.090731000	2.140967000	0.577616000
O	4.627065000	0.008218000	0.487689000
O	-3.685268000	1.234953000	-0.118067000
H	-4.585747000	1.067895000	-0.444887000

Au^+-Tyrosine(-H^+)

N	2.312926000	0.567899000	-1.095619000
C	2.419638000	0.699139000	0.386351000
Au	0.737521000	-0.902167000	-1.503652000
C	1.131420000	1.201477000	1.101851000
C	2.955504000	-0.632872000	0.878613000
C	-0.293146000	-0.770986000	0.505137000
C	-0.131629000	0.616016000	0.538037000
O	3.563788000	-1.406605000	0.176187000
C	-1.094587000	-1.410318000	-0.528396000
O	2.766551000	-0.813909000	2.196230000
C	-0.895917000	1.391116000	-0.370820000
C	-2.051147000	-0.600310000	-1.319991000
C	-1.772720000	0.835062000	-1.276006000
O	-2.988178000	-1.091843000	-1.953846000
H	3.225063000	0.289998000	-1.457026000
H	2.066965000	1.467578000	-1.504842000
H	3.221841000	1.411589000	0.632694000
H	1.271905000	0.989150000	2.164449000
H	1.097486000	2.290206000	0.998413000
H	0.241080000	-1.399150000	1.215163000
H	-1.336432000	-2.465311000	-0.433482000
H	3.212331000	-1.651353000	2.418824000
H	-0.758428000	2.471323000	-0.366709000
H	-2.388784000	1.466046000	-1.909549000

Au_2-Tyrosine(-H^+)

N	-1.290824000	-1.293710000	1.185498000
C	-1.491742000	0.003077000	0.567396000
C	-2.955133000	0.189369000	0.288967000
O	-3.738529000	-0.690411000	0.007848000
H	-0.295401000	-1.507977000	1.147520000
H	-1.769256000	-1.995761000	0.626071000
H	-1.164689000	0.784913000	1.257814000
C	-0.739101000	0.209398000	-0.775136000
C	0.733408000	-0.000519000	-0.600016000
H	-0.951492000	1.218335000	-1.146124000
H	-1.140862000	-0.508136000	-1.500300000

C	3.525056000	-0.450646000	-0.128077000
C	1.541710000	0.990051000	-0.027674000
C	1.342419000	-1.216049000	-0.929843000
C	2.697372000	-1.441937000	-0.706350000
C	2.894464000	0.775958000	0.199988000
H	1.103151000	1.951152000	0.236105000
H	0.743894000	-2.001732000	-1.387004000
H	3.151670000	-2.390225000	-0.979363000
H	3.511807000	1.555617000	0.635722000
O	4.805917000	-0.591916000	0.110031000
O	-3.314568000	1.497850000	0.303138000
H	-4.248142000	1.506853000	0.030267000
Au	5.776619000	-2.321102000	-0.361357000
Au	7.066310000	-4.374738000	-0.897906000

Valine

N	1.139859000	0.846607000	-1.127331000
C	0.176995000	0.873643000	-0.040796000
C	0.831011000	1.045821000	1.316158000
O	2.006485000	0.883858000	1.553649000
H	1.480800000	1.786626000	-1.301318000
H	1.953845000	0.314755000	-0.829773000
H	-0.499231000	1.721557000	-0.186974000
C	-0.683391000	-0.406755000	-0.024667000
H	-1.339391000	-0.338117000	0.848916000
C	0.183658000	-1.654264000	0.105306000
C	-1.540892000	-0.472147000	-1.281296000
H	-0.907523000	-0.487317000	-2.169040000
H	-2.206779000	0.391305000	-1.351551000
H	-2.157236000	-1.373713000	-1.277670000
H	0.812860000	-1.776265000	-0.779203000
H	-0.444442000	-2.543214000	0.187997000
H	0.831683000	-1.620233000	0.984634000
O	-0.069534000	1.377397000	2.270047000
H	0.431223000	1.430457000	3.102912000

Au^+-Valine

N	1.554070000	1.026265000	-0.507165000
C	0.283231000	0.953922000	0.260753000
C	0.560148000	1.175864000	1.735565000
O	1.646893000	1.074517000	2.268625000
H	1.819505000	2.003677000	-0.638958000
H	1.392625000	0.643682000	-1.439709000
H	-0.373897000	1.760273000	-0.080406000
C	-0.448407000	-0.384138000	0.047542000
H	-1.382496000	-0.270393000	0.605524000
C	0.301189000	-1.589952000	0.605366000
C	-0.797010000	-0.582581000	-1.424286000
H	0.085499000	-0.799451000	-2.034921000
H	-1.310238000	0.285274000	-1.844339000
H	-1.457436000	-1.443325000	-1.534237000
H	1.190244000	-1.825740000	0.013823000

H	-0.342675000	-2.469542000	0.560881000
H	0.597593000	-1.458644000	1.648816000
O	-0.566215000	1.465756000	2.373206000
H	-0.364962000	1.570158000	3.322952000
Au	3.168650000	0.093640000	0.420645000

Au_2-Valine

N	0.448351000	1.904125000	0.673588000
C	0.100015000	0.471234000	0.752510000
C	1.112326000	-0.324041000	-0.045077000
O	1.687733000	0.100064000	-1.022413000
H	1.352980000	2.047340000	1.123038000
H	0.598910000	2.137308000	-0.309564000
H	0.150428000	0.168887000	1.800549000
C	-1.332042000	0.233702000	0.240850000
H	-1.965286000	0.813518000	0.921821000
C	-1.729350000	-1.234545000	0.338183000
C	-1.540244000	0.764989000	-1.172003000
H	-0.874026000	0.276723000	-1.888024000
H	-1.391111000	1.844804000	-1.233744000
H	-2.567714000	0.569795000	-1.484028000
H	-1.204896000	-1.843223000	-0.401195000
H	-2.799627000	-1.334146000	0.151207000
H	-1.517527000	-1.648804000	1.325860000
O	1.298878000	-1.558816000	0.452757000
H	1.936721000	-1.999774000	-0.137589000
Au	-0.948839000	3.197973000	1.557981000
Au	-2.601535000	4.698493000	2.595450000
